# Transcriptome-Wide Identification of Differentially Expressed Genes in *Solanum lycopersicon* L. in Response to an *Alfalfa*-Protein Hydrolysate Using Microarrays

**DOI:** 10.3389/fpls.2017.01159

**Published:** 2017-07-05

**Authors:** Andrea Ertani, Michela Schiavon, Serenella Nardi

**Affiliations:** ^1^Department of Agronomy, Food, Natural Resources, Animals, and Environment, University of PadovaPadua, Italy; ^2^Biology Department, Colorado State University, Fort CollinsCO, United States

**Keywords:** biostimulant, productivity, microarray, hormones, phenols, sugars, defense, signaling

## Abstract

An *alfalfa*-based protein hydrolysate (EM) has been tested in tomato (*Solanum lycopersicon* L.) plants at two different concentrations (0.1 and 1 mL L^-1^) to get insight on its efficacy as biostimulant in this species and to unravel possible metabolic targets and molecular mechanisms that may shed light on its mode of action. EM was efficient in promoting the fresh biomass and content in chlorophyll and soluble sugars of tomato plants, especially when it was applied at the concentration of 1 mL L^-1^. This effect on plant productivity was likely related to the EM-dependent up-regulation of genes identified via microarray and involved in primary carbon and nitrogen metabolism, photosynthesis, nutrient uptake and developmental processes. EM also up-regulated a number of genes implied in the secondary metabolism that leads to the synthesis of compounds (phenols and terpenes) functioning in plant development and interaction with the environment. Concomitantly, phenol content was enhanced in EM-treated plants. Several new genes have been identified in tomato as potential targets of EM action, like those involved in detoxification processes from reactive oxygen species and xenobiotic (particularly glutathione/ascorbate cycle-related and ABC transporters), and defense against abiotic and biotic stress. The model hypothesized is that elicitors present in the EM formulation like auxins, phenolics, and amino acids, may trigger a signal transduction pathway via modulation of the intracellular levels of the hormones ethylene, jasmonic acid and abscissic acid, which then further prompt the activation of a cascade events requiring the presence and activity of many kinases and transcription factors to activate stress-related genes. The genes identified suggest these kinases and transcription factors as players involved in a complex crosstalk between biotic and abiotic stress signaling pathways. We conclude that EM acts as a biostimulant in tomato due to its capacity to stimulate plant productivity and up-regulate stress-related responses. Its use in agricultural practices may reduce the need of inorganic fertilizers and pesticides, thereby reducing the environmental impact of productive agriculture.

## Introduction

For a long time, the application of synthetic fertilizers and pesticides has been a common practice to boost crop yield (Calvo et al., 2014; Nardi et al., 2016; Posmyk and Szafrańska, 2016). The main purposes of using these chemical additives in agriculture are the improvement of nutrient supply in soil, crop protection and disease control. Unfortunately, these practices are often responsible for chemical and biological degradation of soil, as a result of undesirable levels of accumulated chemicals (Francioso et al., 2000).

In the last years, there has been a rise in attention brought to the area of agricultural biostimulants (Sharma et al., 2014; Brown and Saa, 2015; Posmyk and Szafrańska, 2016). According to the European Biostimulants Industry Council (European Biostimulants Industry Council [EBIC], 2012a), they are defined as products containing substances and/or microorganisms whose function when applied to plants or the rhizosphere in little amounts is to stimulate natural processes, to enhance/benefit nutrient uptake and use efficiency, tolerance to abiotic stress, and crop quality.

Biostimulants pose an innovative solution to an increased world demand for high crop productivity with less unsustainable inputs (Nardi et al., 2016). They do not fall within the regulatory framework of pesticides (European Biostimulants Industry Council [EBIC], 2012a) and operate in plants through different mechanisms than fertilizers, regardless of the presence of nutrients in the products (European Biostimulants Industry Council [EBIC], 2012b). In this respect, the Biostimulant Coalition (2013) in North America specifies that these products are not nutrients.

Biostimulants are manufactured starting from different sources and include complex mixtures of active substances. Therefore, the assignment of specific functions in plants to their individual components is often arduous. du Jardin (2015) divides biostimulants into a few main categories: humic substances (HS), seaweed extracts, protein hydrolizates and microbial inoculants (mycorrhizal fungi and rhizobacteria). Among them, HS and seaweed extracts are the most extensively studied.

HS include humic and fulvic acids, and consist of relatively small molecules of amphiphilic nature, which can form molecular aggregates or supramolecular associations in solution and on mineral surfaces (Dell’Agnola and Nardi, 1987; Wershaw, 1999; Schaumann, 2006). The effects of HS in plants depend on their concentration, molecular weight, chemical-physical properties, as well as on their hormone-like activity (Dell’Agnola and Nardi, 1987; Nardi et al., 1994; Mora et al., 2012; Ertani et al., 2013; Baglieri et al., 2014; Massa et al., 2016) and capacity to stimulate plant nitrogen (N) assimilation (Quaggiotti et al., 2004). HS generally stimulate root growth, especially in the early stages of plant development (Canellas et al., 2002; Zandonadi et al., 2007; Vaccaro et al., 2009; Mora et al., 2010). They also control nutrient availability via enhancement of root ATPase activity (Maggioni et al., 1987; Nardi et al., 1991; Zandonadi et al., 2010), carbon (C) and oxygen (O_2_) exchange between soil and atmosphere, and can accelerate development cycles (Eyheraguibel et al., 2008). A cDNA-AFLP-based transcriptome study indicated that a broad number of genes involved in developmental and metabolic processes, transcription regulation or RNA metabolism are HS-regulated (Trevisan et al., 2011), while a more recent microarray study revealed that molecular targets of HS in plants are genes related to N, C, and sulfur (S) metabolisms (Jannin et al., 2012).

The other wide category of biostimulants encompasses a variety of seaweed extracts, which are employed by virtue of their high content in bioactive compounds. Phytohormones, such as auxins or cytokinins contained in these extracts, are likely responsible for their action in accelerating and improving plant development (Crouch and Van Staden, 1992; Sivasankari et al., 2006; Khan et al., 2009; Roussos et al., 2009). Effects of seaweeds in plant metabolism include enhancement of chlorophyll content (Mancuso et al., 2006; Sivasankari et al., 2006; Spinelli et al., 2010), earlier germination, flowering and fructification (Sivasankari et al., 2006; Roussos et al., 2009), and higher proliferation of secondary roots (Mugnai et al., 2008; Rayorath et al., 2008; Spinelli et al., 2010). Seaweed-based biostimulants can also induce immunity/resistance to pathogens in plants (Joubert and Lefranc, 2008).

Recently, a microarray based-transcriptomic study has evidenced the activation of both nitrogen and sulfur assimilation pathways in *Brassica napus* plants treated with an *Ascophyllum nodosum* seaweed extract (Jannin et al., 2013). Later, the application of this extract to *B. napus* was found to up-regulate the expression of genes coding for specific transporters, like nitrate (NRT1.1 and NRT2.1) sulfate (SULTR1.1 and SULTR1.2), copper (COPT2) and iron transporters (IRT1), or more generic ones (such as NRAMP3), causing the increase in mineral concentrations in the plant (Billard et al., 2014).

Protein hydrolysates represent another notorious category of biostimulants, which consists of amino acids and peptide mixtures. These components are manufactured through chemical and/or enzymatic protein hydrolysis using agroindustrial by-products, from both plant sources (crop residues) and animal wastes (i.e., collagen, epithelial tissues) (du Jardin, 2015). Biostimulants properties of protein hydrolysates are mainly ascribable to their content in low molecular fraction (Quartieri et al., 2002) and free amino acids (Cavani and Ciavatta, 2007).

Previous studies showed that a fabaceae (*alfalfa*) plant derived-protein hydrolysate stimulated maize plant growth by fostering the activity and gene expression of several enzymes involved in nitrogen (N) assimilation and carbon (C) metabolism (Schiavon et al., 2008; Ertani et al., 2009, 2013a), while phenol-containing protein hydrolysates enhanced phenylpropanoid metabolism in the same species (Ertani et al., 2011b). Recently, a metabolic approach highlighted the role of protein hydrolysates in increasing the activity of the light reactions components and Calvin cycle enzymes, and in the promotion of antioxidant accumulation (Ertani et al., 2014).

Despite these described effects of protein hydrolysates in plants, many other still remain to unravel as compared to other classes of biostimulants, especially with respect to the molecular mechanisms triggered by them in aiding plants to overcome stressful conditions. Only recently, a transcriptome study performed in maize plants showed that targets of protein hydrolysates are genes related to cell wall organization, transport processes, stress responses and hormone metabolism (Santi et al., 2017).

Therefore, there is a clear need to enrich our understanding of protein hydrolysate function and mechanism of action in crops in order to develop more efficient materials to apply to plants growing under unfavorable or suboptimal conditions and optimize the industrial processes employed for their manufacturing.

Because transcriptome approaches would be functional to determine the metabolic targets of protein hydrolysates in plants and highlight the signaling pathways involved in the responses of crops to biotic and abiotic stresses, cDNA microarray has been used in this study as a quantitative method for global and simultaneous analysis of plant gene expression to gain knowledge about the expression profiles of genes involved in the responses of the crop tomato (*Lycopersicon esculentum*, cv Microtom) to an *alfalfa*-derived protein hydrolysate.

## Materials and Methods

### Chemical and Spectroscopic Characterization of the Biostimulant

The biostimulant used in the current study, EM, was manufactured by ILSA S.p.A. (Arzignano, VI, Italy) and produced through a fully controlled enzymatic hydrolysis using *alfalfa* (*Medicago sativa* L.) plants. The chemical, physical and spectroscopic characterization of this alfalfa hydrolysate has been previously described by Schiavon et al. (2008) and Ertani et al. (2014).

### Experimental Design and Plant Growth

To estimate the effects of EM on tomato plants, a hydroponic experiment was performed. Seeds of tomato (*Solanum lycopersicon* cv. Microtom) were surface-sterilized by rinsing in 70% (v/v) ethanol for 30–60 s, then in 5% (v/v) sodium hypochlorite (NaClO) for 30 min while rocking on a platform, and washed in distilled water for 5 × 10 min. The seeds were allowed to germinate and grow for 12 days in half-strength MS (Murashige and Skoog, 1962) agar medium inside a growth chamber with a 14 h light/10 h dark cycle, air temperature of 26/21°C, relative humidity of 70/85% and at a photon flux density (PFD) of 280 mol m^-2^ s^-1^. Germinated seedlings were transferred to 3 L pots (density = 6 plants per pot) and cultivated in a thoroughly aerated Hoagland and Arnon (1950) modified nutrient solution with the following composition (μM): KH_2_PO_4_ (80), Ca(NO_3_)_2_ (1000), KNO_3_ (250), MgSO_4_ (1000), FeNaEDTA (20), B (4.6), Cl (1.1), Mn (0.9), Zn (0.09), and Mo (0.01). The nutrient solution in each pot was renewed every 3 days to ensure a constant supply of macro- and microelements to plants. After 25 days since the transplant, the EM protein hydrolysate was added to the nutrient solution for 48 h at the following concentrations: 0.1 and 1 mL L^-1^. These doses and the exposure time were selected based on previous studies screening the most efficient EM concentration in inducing positive physiological responses in plants (Schiavon et al., 2008; Ertani et al., 2009). A group of plants that was not treated with the biostimulant was used as the control. At the end of 48 h, plants were harvested and used for all further molecular and physiological analyses. For fresh weight measurements, 10 plants per treatment were divided into roots and shoots and weighed separately. Samples from the remaining plants were immediately frozen with liquid nitrogen after harvest and kept at -80°C for further analyses.

The experimental design was factorial (species × biostimulant concentration) with 4four replicates (1 replicate (=1 pot with 6 plants each) per treatment. All plants collected were representative of the peculiar traits of the species.

### Determination of Chlorophyll Content

The quantification of relative chlorophyll concentration was performed through a non-destructive method that uses light transmission through a leaf, at two wavelengths, to determine the greenness and thickness of leaves. Transmission in the infrared range provides a measurement related to leaf thickness, and a wavelength in the red light range is used to determine greenness. The ratio of the transmission of the two wavelengths provides a chlorophyll content index that is referred to as a Soil Plant Analysis Development (SPAD) index (Richardson et al., 2002).

A SPAD (Soil Plant Analysis Development) chlorophyll meter (SPAD-502 model, Minolta Camera Co., Ltd., Osaka, Japan) was used to measure the SPAD index on the last expanded leaf of tomato plants. The determination was carried out on 5 measurements per leaf from 10 plants per experimental condition.

### RNA Extraction and Purification

RNA extraction from three biological replicates of leaves and roots was performed using a phenol/chloroform protocol according to Sambrook and Russell (2001). After RNA extraction, DNase treatment was applied (DNase1, Sigma–Aldrich), following the manufacturer’s instruction. RNA quality was confirmed by agarose gel electrophoresis, and the concentration and purity of the RNA samples were assessed using a NanoChip Agilent 2100 BioAnalyzer (Agilent, Santa Clara, CA, United States).

### Preparation of Fluorescent Dye-Labeled cDNA and Hybridization

Briefly, 100 ng of purified RNA was reverse transcribed according to the manufacturer’s instructions using 200 U of Superscript Reverse Transcriptase III (Life Technologies). Controls and treated samples were compared and, respectively, labeled with fluorescent dyes cyanine 3-CTP and cyanine 5-CTP. After the labeling step, cRNA samples were purified using the RNeasy Mini Kit (Qiagen), and then fragmented to take away secondary structures (specific buffer at 60°C for 30 min) enabling cRNA lengths of between 50 and 200 nucleotides to be obtained and then optimal hybridization with an Agilent 60-mer oligonucleotide microarray to be carried out. Thereafter, probe hybridizations were performed at 65°C for 17 h. Each test sample was hybridized using a *Solanum lycopersicon* Gene Expression microarray 4 × 44 K (Agilent Technologies^®^) using a one-color Microarray-Based Gene Expression Analysis (Quick Amp Labeling). All the procedures were performed at CRIBI (Centro di Ricerca Interdipartimentale per le Biotecnologie Innovative, University of Padova, Italy).

### Image Acquisition and Bioinformatic Analysis

After probe hybridization, microarrays were scanned with Agilent Scan Control software using default parameters for 4 × 44 K formats. Data were extracted with Agilent Feature Extraction (FE) program 10.5.1.1 (Agilent Technologies^®^).

Global mean intra-array (Moltiplicatively Detrended) and inter-array (Quantile) normalization was performed across element signal intensity and expression values were transformed into Log_2_ ratio of normalized intensities. For annotation of transcripts an annotated probe file was used as a reference, which was generated at EMBL-EBI (Array Express, A-GEOD-8648-Agilent Custom Tomato Gene Expression 4 × 44 k Array AMADID:19003) and NCBI website.

Significantly up- or down-regulated genes were filtered with fold-change values ≥2 or ≤-2, respectively, with *q*-value ≤ 0.05 in *t*-test. The program Blast2GO 2.8 was used to perform the gene ontology (GO) analysis and cluster genes based on the biological process.

MapMan and PageMan^1^ analyses were done as described in Galla et al. (2009) using the *S. lycopersicon* dataset properly rearranged as input files with the correct genechip identifiers (Agilent) using the pathway dataset Slyc_AGILENT44k _SGN_BUILD2.

Gene sets filtered as explained above were selected for drawing Venn diagrams using the Web-based tool Venn Diagram Generator^2^.

### Validation of Gene Expression by Real-Time Quantitative PCR (qRT-PCR)

For quantitative Real-Time PCR experiments, RNA was extracted from three individual samples of leaves and roots of tomato plants grown in hydroponics under the following experimental conditions: control, plus EM 0.1 mL L^-1^, Se 40 mM. RNA extraction was performed using a phenol/chloroform protocol according to Sambrook and Russell (2001). All the cDNAs were prepared from 3 μg of RNAs using 200 U of ImProm-II^TM^ Reverse Transcriptase (Promega, Milan, Italy) and oligodT as primers in 20 μl reaction volume. Mixtures were incubated at 37°C for 60 min, 70°C for 5 min, and 4°C for 5 min to stop the RT reaction. Specific primer pairs for the selected sequences are reported in Supplementary Table S1 and tested for their activity at Tm ranging from 58 to 65°C by conventional PCR. qRT-PCR analyses were performed using a thermal cycler 7300 Real-Time PCR System (Applied Biosystem) equipped with a 96 well plates system with the SYBR green PCR Master Mix reagent (Applied Biosystem). Each qPCR reaction (10 μl final volume) contained 1 μl of diluted cDNA (1:10), 1 μL of primer couple (10 μM), and 5 μl of 2× SYBR Green PCR Master Mix according to the manufacturer’s instructions. The following thermal cycling profile was used for all PCRs: 95°C for 10 min, 50 cycles of 95°C for 15 s, 60°C for 1 min). The gene expression analysis for each biological replicate was evaluated in two technical replicates (only one set of data is shown in figures). All quantifications were normalized to the actin gene used as housekeeping gene and amplified in the same conditions. Data resulting from qRT-PCR were normalized on the basis of the housekeeping gene by using the ΔΔCt method (Livak and Schmittgen, 2001) and compared to those obtained via microarray. Given the high variation in gene expression, for simplicity data in figures are reported as Log_2_ ratio of normalized intensities.

### Determination of Total Antioxidant Activity, Phenol and Sugar Compounds

The total antioxidant activity (TAC) was evaluated by measuring the ferric-reducing antioxidant power. The assay was based on the methodology of Benzie and Strain (1996). Ten grams of plant material was homogenized in 20 mL of HPLC grade methanol using an Ultra-Turrax tissue homogenizer (Takmar, Cincinnati, OH, United States) at moderate speed (setting of 60) for 30 s. The ferric-reducing antioxidant power (FRAP) reagent was freshly prepared, containing 1 mM 2,4,6-tripyridyl-2-triazine (TPTZ) and 2 mM ferric chloride in 0.25 M sodium acetate buffer at pH 3.6. One hundred microliters of the methanol extract was added to 1,900 μL of FRAP reagent and accurately mixed. After leaving the mixture at 20°C for 4 min, the absorbance was determined at 593 nm. Calibration was against a standard curve (0–1,200 μg mL^-1^ ferrous ion) obtained by the addition of freshly prepared ammonium ferrous sulfate. FRAP values were calculated as microgram per milliliter ferrous ion (ferric-reducing power) and are presented as milligram per kilogram of Fe^2+^ Eq (ferrous ion equivalent).

The concentration of total phenols was determined by the Folin-Ciocalteau (FC) assay with gallic acid as calibration standard, using a Shimadzu UV-1800 spectrophotometer (Shimadzu Corp., Columbia, MD, United States). The FC assay was performed by pipetting 200 μL of plant extract (obtained as described above for sugars analysis) into a 10 mL PP tube. This operation was followed by addition of 1 mL of Folin-Ciocalteau reagent. The mixture was vortexed for 20 to 30 s. Eight hundred microliters of sodium carbonate solution (20% w/v) was added to the mixture 5 min after the addition of the FC reagent. This was recorded as time zero; the mixture was then vortexed for 20 to 30 s after addition of sodium carbonate. After 2 h at room temperature, the absorbance of the colored reaction product was measured at 765 nm. The total phenols concentration in the extracts was calculated from a standard calibration curve obtained with different concentrations of gallic acid, ranging from 0 to 600 μg mL^-1^. Results were expressed as milligrams of gallic acid equivalent per kilogram of fresh weight (Nicoletto et al., 2013).

For the determination of soluble sugars, leaf and root samples (2 g) were homogenized in water (20 mL) with an Ultra Turrax T25 (IKA, Staufen, Germany) at 13,500 rpm until uniform consistency. Samples were filtered with filter paper (589 Schleicher) and further sieved through cellulose acetate syringe filters (0.45 μm). The analysis of the extracts was performed using an HPLC apparatus (Jasco X.LC system, Jasco Co., Tokyo, Japan) consisting of a model PU-2080 pump, a model RI-2031 refractive index detector, a model AS-2055 autosampler and a model CO-2060 column oven. ChromNAV Chromatography Data System was used as software. Sugars were separated on a Hyper-Rez XP Carbohydrate Ca^2+^ analytical column (7.7 mm × 300 mm, Thermo Scientific, Waltham, MA, United States) operating at 80°C. Isocratic elution was performed using water at a flow rate of 0.6 mL/min. The peaks were identified by comparing the retention time with those of standard compounds. To calculate the concentrations in the extract, a calibration curve was drawn for four solutions of known concentration in water.

### Elemental Analysis

Quantification of Fe, K, P, and S in leaves of tomato plants was obtained after an acid digestion procedure using a microwave (Milestone Ethos model 1600, Milestone, Shelton, CT, United States). All digestion reactions were carried out in sealed 120 mL Teflon vessels using 500 mg plant material and 10 mL of 30% (v/v) HCl as a solvent. Digested samples were then diluted in 10 mL ultrapure water and assayed via Inductively Coupled Plasma Atomic Emission Spectroscopy (Spectrum Ciros CCD, Kleve, Germany).

Total nitrogen in leaf tissues was detected through a dry combustion procedure using an element analyzer (vario MACRO CNS, Hanau, Germany).

### Statistical Analysis

For all determinations with the exception of microarray, the analysis of variance (ANOVA) was performed using the SPSS software version 18.0 (SPSS, Chicago, IL, United States), and was followed by pair-wise *post hoc* analyses (Student–Newman–Keuls test) to determine which means differed significantly at *p* < 0.05 (±SD). The number of biological replicates analyzed were three for qRT-PCR and element quantification, 10 for plant growth and SPAD, five for total antioxidant capacity (TAC), phenols and sugars determinations.

For microarray, in each experiment (tissue vs. EM concentration), probes with [Marginal] flag and at least one channel above the background for the three biological replicates were retained. A *t*-test was applied on each filtered gene list according to the following parameters: (i) *t*-test against zero, (ii) Benjamini–Hochberg correction and (iii) *p*-value < 0.05. Only genes whose expression was modified at least by a fold change of 2 (chosen as a threshold) were included in the list of differentially expressed genes (DEGs) (**Table 1** and Supplementary Table S2).

**Table 1:** Partial list of up-regulated genes in leaves and roots of *Solanum lycopersicon* plants treated with either EM 0.1 mL L^-1^ or EM 1 mL L^-1^ by both EM dosages.

Gene name	Agilent ID	Fold change		Annotation
		EM 0.1 mL L^-1^	EM 1 mL L^-1^	
Leaves				
AI485516	A_96_p132312	1170.57	2.43	Basic helix-loop-helix (bHLH)
TA37435	A_96_p011406	265.40	265.31	Aldo/keto reductase
TA56542	A_96_p126097	177.42	1170.18	expansin
AK326750	A_96_p120972	176.80	2.63	Phox (PX) domain-containing protein
AI771499	A_96_p133717	126.68	85.78	AP2 domain-containing transcription factor, putative
AK329872	A_96_p107139	99.59	15.92	Peroxidase, putative
BT012835	A_96_p103444	28.46	115.49	Transferase family protein
TA54953	A_96_p119612	16.83	2.46	Homeobox-leucine zipper
AI487014	A_96_p131332	16.60	2.58	Putative bzip transcription factor
AW029915	A_96_p143491	13.75	2.58	Lactoylglutathione lyase
AK321258	A_96_p171729	11.17	27.52	Cytochrome P450 94A1 (CYP94C1)
AW443470	A_96_p156756	10.51	2.98	Transferase family protein
AK323400	A_96_p036591	6.43	33.07	Homeobox-leucine zipper protein 12 (HB-12)
TA56836	A_96_p127927	4.68	10.69	Zinc finger (Ran-binding)
M61914	A_96_p171334	4.51	18.34	L-threonine ammonia-lyase
AK322433	A_96_p045476	4.07	17.40	Glutathione *S*-transferase
TA56114	A_96_p124337	3.45	16.59	CTF2A monooxygenase
AK326774	A_96_p043686	3.09	10.68	Hydrolase
BE344500	A_96_p014241	3.08	126.69	Alternative oxidase 1A (AOX1A)

**Roots**				

BF097588	A_96_p181989	7.14	3.72	Cell wall-associated hydrolase
AK322433	A_96_p045476	6.91	17.89	Glutathione *S*-transferase
TA37435	A_96_p011406	6.65	2.69	Aldo/keto reductase family protein
TA38046	A_96_p181024	6.09	2.43	Heat shock protein 91
BG130524	A_96_p187884	5.94	2.31	Chitinase
AK328987	A_96_p151561	5.28	6.98	Calcium-dependent protein kinase 33
BI933689	A_96_p206279	5.25	2.36	Aldo/keto reductase family protein
AW041795	A_96_p059781	4.86	3.29	Ribosomal protein L7Ae family protein
BI923348	A_96_p102244	4.52	2.32	Multidrug resistance-associated protein 6
DV104033	A_96_p246567	4.09	18.34	Chitinase
TA50778	A_96_p108952	4.05	5.23	Calcium-dependent protein kinase 33
AW224326	A_96_p155271	3.94	21.21	Alcohol dehydrogenase 1
CK468693	A_96_p054591	3.12	11.63	Chromosome chr8 scaffold_23, transcription factor
BI421662	A_96_p043431	2.73	85.78	Expansin-Like B1
DB703551	A_96_p232879	2.58	6.42	Kelch repeat-containing protein type 1
AK328356	A_96_p008426	2.44	9.55	Universal stress protein (USP) family protein

*Annotation is given based on blast search against the plant sequence database. Selection for sequences is based on fold change (>10 and >5 for leaves and roots, respectively, for at least one treatment).*

## Results

### Plant Growth and Chlorophyll Determination

**FIGURE 1:**
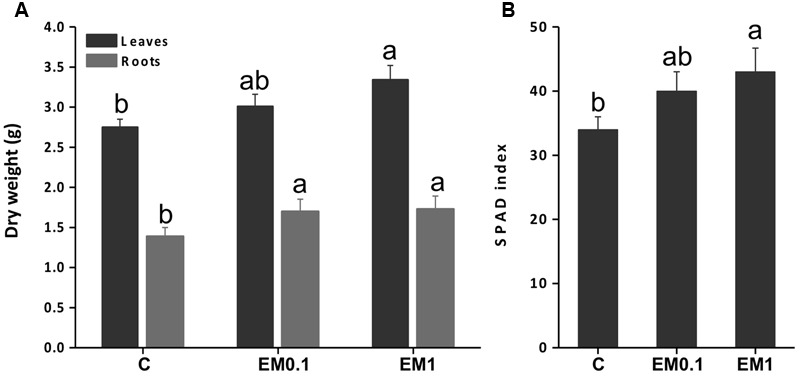
Leaf and root fresh weight **(A)** and Soil Plant Analysis Development (SPAD) index **(B)** of tomato (*Solanum lycopersicon*) plants grown in the absence of EM (control, C), and in the presence of either EM at 0.1 mL L^-1^ or 1 mL L^-1^. Values are expressed per plant. Letters above bars indicate significant differences between treatment (*P* < 0.05; *n* = 10).

The application of the biostimulant EM to tomato plants at either 0.1 ml L^-1^ or 1 ml L^-1^ concentration led to higher leaf and root fresh biomass production (**Figure 1A**). In leaves, the increase was more pronounced (plus 37%) when plants were supplied with EM 1 ml L^-1^, while root growth was improved equally (plus 21.4%) by the two EM doses.

The analysis of the SPAD index was conducted to verify the effectiveness of the biostimulant EM in improving tomato plant productivity via enhanced chlorophyll synthesis. The results obtained indicated that SPAD index was increased by EM treatment in a dose dependent manner, being the maximum values recorded in plants added with EM 1 ml L^-1^ (**Figure 1B**).

### Transcriptome Analysis Overview

**FIGURE 2:**
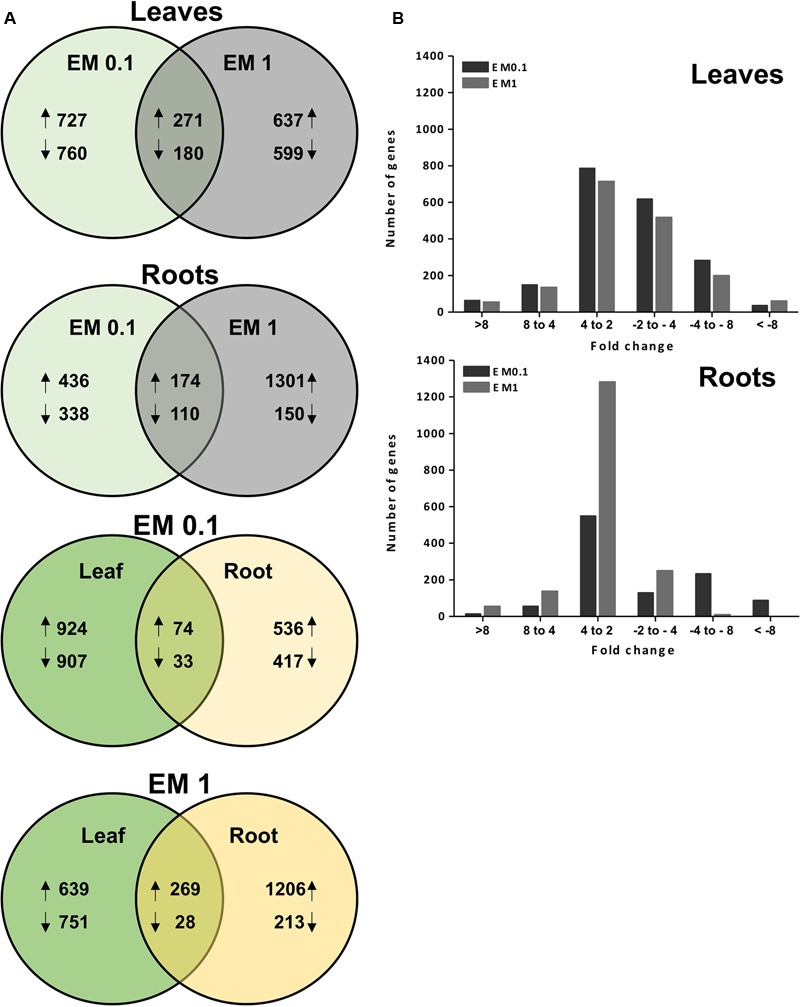
**(A)** Venn diagram analysis for the EM-responsive genes under the different EM treatments in leaves and roots of *S. lycopersicon*. Overlapping circles visually represent the commonalities among sets of information. In the first two upper diagrams, arrows upward and downward indicate up-regulation and down-regulation, respectively, compared to the control (no EM treatment). The number of differentially expressed (DEGs) relative to a specific ET treatment (EM at 0.1 mL L^-1^ or EM 1 mL L^-1^) is displayed in the non-overlapping regions of the circles. In the lower two diagrams, the number of sequences commonly or individually up-regulated or down-regulated in leaves and roots at a specific EM dosage **(B)**. Distribution of DEGs in leaves and roots of *S. lycopersicon* based on fold-changes values. The positive and negative numbers on the x-axis represented up- and down-regulation of *S. lycopersicon* genes, respectively.

The microarray study was aimed at profiling the EM-responsive genes in leaves and roots of tomato plants treated for 2 days with EM at two different dosages. A total of about 32,000 genes were assayed per each plant tissue × EM treatment. The transcript level of 1938 genes in leaves and 1054 genes in roots of plants supplied with EM 0.1 ml L^-1^ was significantly altered compared to the control plants, with fold-change values ≥ 2 or ≤-2 (*q*-value < 0.05) (**Figure 2A**). Based on the same fold change intervals, the transcript abundance of 1687 genes in leaves and 1735 genes in roots of plants supplemented with EM 1 ml L^-1^ showed significant variation (**Figure 2A**).

Of these genes, 271 and 174 were up-regulated by both EM dosages in leaves and roots, respectively, while 180 and 110 were down-regulated. However, a large number of genes was found to be regulated by a definite EM treatment. Specifically, in leaves the transcript level of 727 and 637 transcripts was increased by EM 0.1 ml L^-1^ and EM 1 ml L^-1^, respectively, while 760 and 599 were reduced in expression. In roots, the number of differential expressed genes (DEGs) was 774, of which 436 up-regulated and 338 down-regulated, when plants were supplied with EM 0.1 ml L^-1^. The number of root DEGs was twofold higher in plants added with EM 1 ml L^-1^ and included 1301 up-regulated and 150 down-regulated transcripts.

In the case of sequences that showed a similar trend of regulation between leaves and roots for each test biostimulant dose, we observed that 74 and 269 were up-regulated in plants treated with EM 0.1 and EM1, respectively, while 33 and 28 were down-regulated (**Figure 2A**).

Despite the high number of identified DEGs, only part of them had reported homologs or showed homology to genes coding for predicted proteins (*E*-value < 1.0 E^-5^) using the blastx program against the plant nr (NCBI) database. These DEGs are listed in **Table 1** (partial list) and Supplementary Table S2 (complete list). The remaining DEGs corresponded to proteins of unknown function or uncharacterized. The fold-change based-distribution of genes significantly altered in expression by EM indicates that in leaves these sequences were mainly within the fold-change range of +2 to +4 when up-regulated, and in the range of -2 to -4 if down-regulated, regardless of the EM dose. In roots, most of sequences fell in the range of +2 to +4, while a minor number was assigned to other ranges (**Figure 2B**).

**FIGURE 3:**
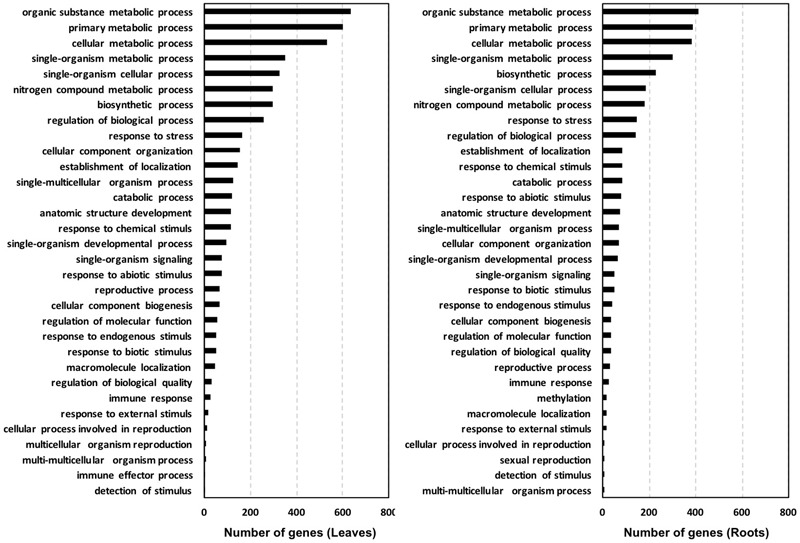
Gene ontology (GO) classification of the probes used in the tomato cDNA microarray with significant similarity in NCBI protein databases. Classification is shown based on biological process using the software Blast2GO (cutoff 3mp).

Based on the biological process, the GO classification of the probes used in cDNA microarray arranged the DEGs in a few prominent categories shared by leaves and roots, with a sequence number >150 each (**Figure 3**). These categories included: organic substance metabolic process, primary metabolic process, cellular metabolic process, single-organism metabolic process, biosynthetic process, nitrogen compound metabolic process, response to stress and regulation of biological process. Other biological processes in which DEGs were entered and accounted for less than 150 sequences each are shown in **Figure 3**.

**FIGURE 4:**
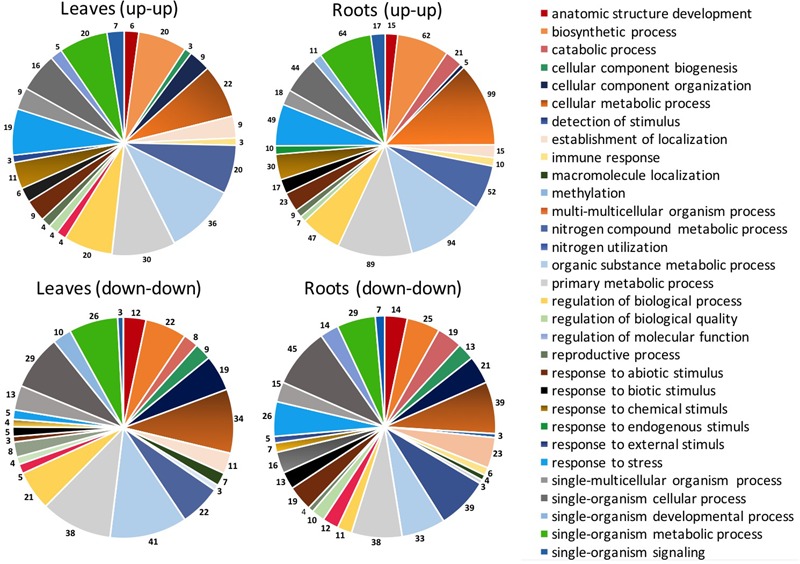
Distribution of DEGs based on biological process for sequences up-regulated or down-regulated in leaves and roots of *S. lycopersicon* by both EM 0.1 mL L^-1^ and EM 1 mL L^-1^. The different categories reported on the right side are referred to the color of circle slices starting from the red category (anatomic structure development) and moving according to clockwise. Only categories with sequence number >3 are shown.

In general, the total number of sequences belonging to the main biological process categories was higher for leaves compared to roots, especially with respect to organic substance metabolic process (+54%), primary metabolic process (+22%), and cellular metabolic process (+10%). This trend was mainly ascribed to sequences regulated by a definite EM treatment, as the specific distribution of genes up-regulated by both EM dosages in leaves and roots of *S. lycopersicon* plants indicated that sequences involved in these processes are more represented in roots than in leaves, while no significant differences in number of down-regulated sequences were evident between leaves and roots (**Figure 4**).

### Identification of Metabolic Pathways Regulated by EM

The genes that were identified as differentially expressed in microarray were mapped into functional groups via MapMan in order to gain insight into which gene families and metabolic pathways may represent targets of regulation by EM.

**FIGURE 5:**
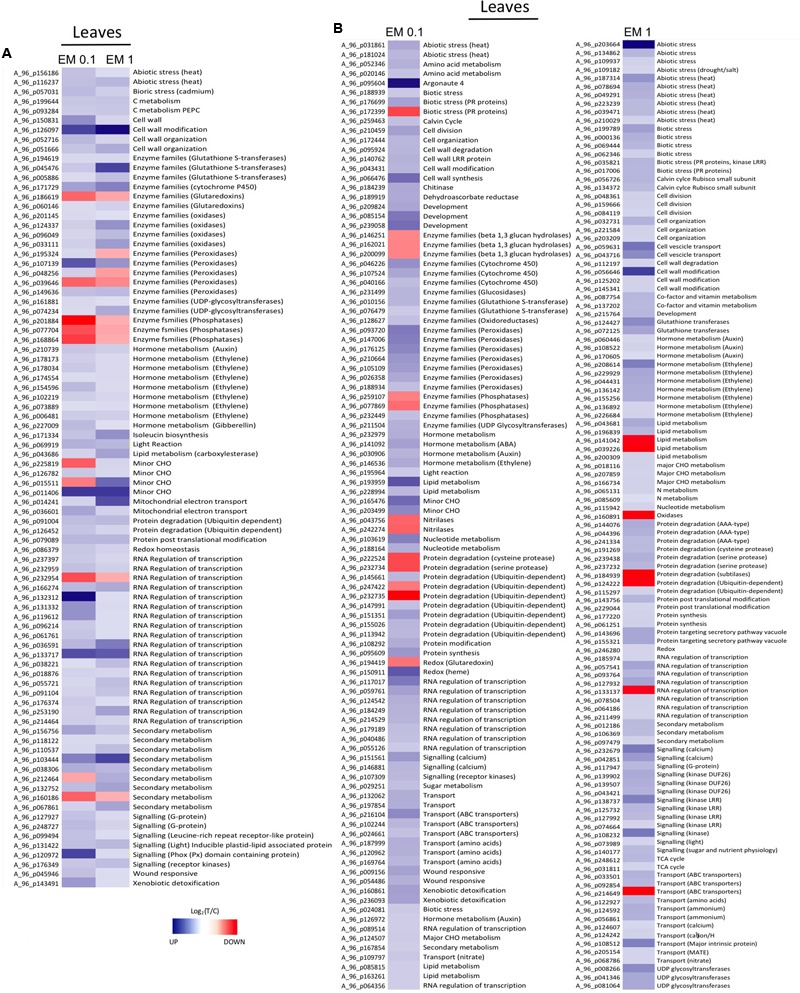
**(A)** Heat Map of representative sequences differentially expressed in leaves of *S. lycopersicon* plants by both EM dosages (0.1 mL L^-1^ and EM 1 mL L^-1^). **(B)** Heat Map of representative sequences that showed differential expression in leaves of *S. lycopersicon* treated with a definite EM dosage. Specifically, on the left are the sequences differentially expressed only under EM 0.1 mL L^-1^, on the right are sequences differentially expressed only under EM 1 mL L^-1^. The corresponding metabolic pathways associated to the sequences are reported on the right side, while the Agilent codes are shown on the left side of each column. Different tones of blue and red indicate up-regulation and down-regulation, respectively.

**FIGURE 6:**
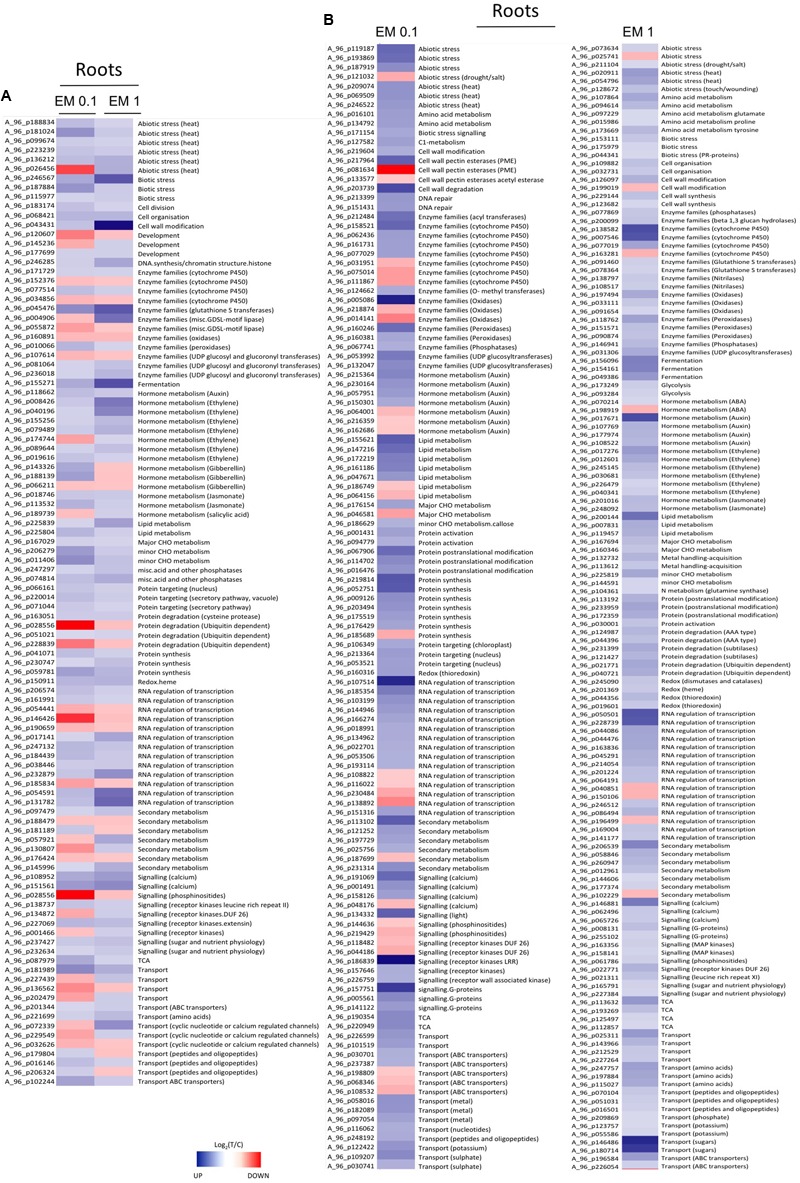
**(A)** Heat Map of representative sequences differentially expressed in roots of *S. lycopersicon* plants by both EM dosages (0.1 mL L^-1^ and EM 1 mL L^-1^). **(B)** Heat Map of representative sequences that showed differential expression in leaves of *S. lycopersicon* treated with a definite EM dosage. Specifically, on the left are the sequences differentially expressed only under EM 0.1 mL L^-1^, on the right are sequences differentially expressed only under EM 1 mL L^-1^. The corresponding metabolic pathways associated to the sequences are reported on the right side, while the Agilent codes are shown on the left side of each column. Different tones of blue and red indicate up-regulation and down-regulation, respectively.

From **Figures 5, 6**, as well as from Supplementary Table S2, it is evident that some gene families are prominently present. They include defense-related genes [particularly Cytochrome 450 (Cyt450), leucine rich repeat proteins (LRR), heat shock proteins (HSP), aldo/keto reductase, glutathione-*S*-transferases (GSTs), lactoylglutathione lyase, DNAJ, chitinases, pathogenesis-related protein Bet v, subtilases, DREB2A, hydroxyproline-rich glycoprotein, wound-induced proteins, L-threonine ammonia-lyase, alternative oxidase 1A, syntaxin (SYR1), DC1 domain-containing proteins, thaumatin, ECERIFERUM (CER1), serine carboxypeptidase-like, aspartyl protease, CC-NBS-LRR proteins]; antioxidant-related genes [mainly peroxidases including ascorbate peroxidase (APX), catalases, thioredoxins, hemoglobins, glutaredoxins GRX, dehydroascorbate reductase (DHAR), CTF2A monooxygenases]; transcription factors [e.g., basic helix-loop-helix (bHLH), AP2/EREBPs (APETALA2), homeobox-leucine zipper, bzip, zinc finger (ZFN), pentatricopeptide repeat-containing protein (PPR), WRKY, Myb, Rav, ERF2, chromosome chr8 scaffold_23]; protein kinases [particularly MAPK, MAPKKK, diacylglycerol kinase, calcium and/or calmodulin-domain protein kinases, serine/threonine-protein kinases, CBL-interacting protein kinases, wall-associated kinase 2 (WAK2), cyclin-dependent protein kinases, S-locus lectin protein kinases]; nitrate metabolism genes [particularly nitrate reductase (NR), aspartate aminotransferase (AST), glutamine-dependent asparagine synthetase (ASN1), glutamine synthetase (GS)]; sugar and lipid metabolism-related genes (e.g., lipases, polygalacturonases, pectinesterases, starch synthase, sucrose synthase, cellulose synthase, inositol oxygenases); hormone-related genes [ethylene forming enzyme (EFE), 1-aminocyclopropane-1-carboxylate oxidase (ACO), ein3-binding f-box protein 1, jasmonic acid-amino acid-conjugating enzyme, protein phosphatase 2C (PP2C), ethylene/ auxin/ABA/gibberellin- responsive proteins, S-adenosyl-l-methionine:salicylic acid carboxyl methyltransferase]; photosynthesis-related genes [particularly Ribulose bisphosphate carboxylase, ferredoxin, phytochrome interacting factor 3-like 5 (PIL5), photosystem II 22 kda protein]; development-related genes [mainly expansins, growth-regulating factors 3 (GRF3) and 5 (GRF5), embryo defective proteins, lob domain protein 1]; secondary metabolism-related genes [mainly phenylalanine ammonia lyase (PAL), 2-oxoglutarate-dependent dioxygenase, hydroxy-3-methylglutaryl coenzyme A reductase, isoflavone reductase, terpene synthase, caffeoyl-CoA 3-*O*-methyltransferase], protein degradation (particularly F-box, ubiquitin and ubiquiting conjugating enzymes, cysteine proteases, AAA-ATPase).

With respect to genes involved in the transmembrane transport of substances, the most represented were those coding for amino acid and peptide transporters, ABC transporters, MATE efflux transporters, tonoplast intrinsic proteins (TIP), ATPases. Other transporters of interest up-regulated under EM treatment were sulfate transporters SULTR 2;1 and SULTR 3;1, nitrate transporter NTR2, ammonium transporter AMT1.1, copper transporters, phosphate transporter PT2, iron-phytosiderophore transporter protein yellow stripe 1 (YS1), potassium channels, sugar transporter, auxin transporter (zinc induced facilitator, ZIFL1), organic cation/carnitine transporters, nodulin MtN21, and glutathione-conjugate transporter MRP4.

A number of GTP-binding proteins involved in protein synthesis and intracellular translocation, as well as UDP glucosyltransferases were also up-regulated by both EM dosages, regardless of the plant organ. Interestingly, two genes, one coding for a polypyrimidine tract-binding protein (PTB) and the other one for a RNA recognition motif (RRM)-containing protein, were strongly up-regulated with a fold change of 626 and 32, respectively, in leaves of plants treated with EM 0.1 ml L^-1^. Both genes play a role in splicing, mRNA stability and translation initiation. Genes coding for RNA recognition motif (RRM)-containing proteins were also identified in leaves of plants supplied with EM 1 ml L^-1^, however, the fold change values were much lower (barely above 2).

### Comparison of DEGs Regulated by Different EM Dosages

The complete list of DEGs that matched with known proteins is reported in Supplementary Table S2, while a partial list of up-regulated genes by both EM concentrations is shown in **Table 1**. Considering the DEGs that are strongly up-regulated (fold change >10) in leaves of *S. lycopersicon* plants by both EM dosages, we identified only the genes encoding for aldo/keto reductase and AP2 domain-containing transcription factor with a comparable fold change value (Supplementary Table S2). Genes encoding for a bHLH transcription factor, a phox (PX) domain-containing protein, a peroxidase, a homeobox-leucine zipper protein, a putative bzip transcription factor, a lactoylglutathione lyase were prominently up-regulated by EM 0.1 ml L^-1^. As an example, the fold changes for the bHLH transcription factor and phox (PX) domain-containing protein were 1170.6 and 176.8, respectively, vs. the 2.43 and 2.63 values determined under EM 1 ml L^-1^ application. However, a number of DEGs was significantly more expressed in leaves of plants treated with the highest EM dose, such as an expansin, a Cytochrome P450 94A1, a homeobox-leucine zipper protein 12 (HB-12), a zinc finger (Ran-binding) protein, a glutathione *S*-transferase, a CTF2A monooxygenase, an alternative oxidase 1A. The remaining DEGs shared fold change values of comparable magnitude.

With respect to the DEGs whose expression was increased by both EM concentrations in roots, a similar trend for a few of the genes previously mentioned was observed. For instance, the aldo/keto reductase encoding gene displayed a higher fold change value in roots of plants treated with EM 0.1 ml L^-1^, while glutathione *S*-transferase and expansin genes were more strongly up-regulated by EM 1 ml L^-1^. The genes encoding for chitinases, chromosome chr8 scaffold_23 transcription factor and alcohol dehydrogenase 1 were also more expressed under the EM 1 ml L^-1^ treatment.

In the case of DEGs regulated by a definite EM treatment, 218 and 481 with known function were up-regulated in leaves by EM 0.1 ml L^-1^ and EM 1 ml L^-1^, respectively. The genes induced by EM 0.1 ml L^-1^ with a fold change >10 included a polypyrimidine tract-binding protein, two embryo defectives (EMB1379 and EMB1303), a lipase class 3, a hemoglobin class 1, an RNA recognition motif (RRM)-containing protein, an inositol oxygenase, a cellulose synthase catalytic subunit, a nodulin MtN21 family protein, a zinc finger (CCCH-type) protein, three peroxidases, a ribose-phosphate pyrophosphokinase 2, a putative kiwellin ripening-related protein precursor, and a MATE efflux family protein. Different DEGs with a fold change >10 were up-regulated by EM 1 ml L^-1^ compared to EM 0.1 ml L^-1^. Among these are a pathogenesis-related protein Bet v family, an expansin like B1, an In2-1 protein, an oxidoreductase, a late embryogenesis abundant domain-containing a protein syntaxin-related protein (SYR1), a protein kinase, a CAM1 (CALMODULIN 1), a DC1 domain-containing protein, a 2-oxoglutarate-dependent dioxygenase, a leucine-rich repeat protein, a BETA-TIP (beta-tonoplast intrinsic protein), lob domain protein 1, coatomer protein complex Glutathione *S*-transferase, UDP-glucoronosyl/UDP-glucosyl transferase.

In roots, the fold change determined for genes up-regulated only by EM 0.1 ml L^-1^ was generally lower than 10, with the exception of a leucine-rich repeat protein, whose fold change was 10.2. The other genes that displayed fold change values at least higher than 5 under this EM treatment were an oxidoreductase, a zinc-binding dehydrogenase protein, a PHD zinc finger protein, a protein responsive to abscisic acid 1B (RAB1B), a glycosyl hydrolase. On the contrary, the fold change of genes whose transcript abundance was specifically enhanced by EM 1 ml L^-1^ was higher than 10 for 13 of them, including a zinc induced facilitator-like 1 (ZIFL1), an organic cation/carnitine transporter 2, a Cytochrome P450 94B2, an indolacetic acid (IAA)-amido synthases, a Cytochrome P450 86A7, a LOB domain protein 41, a pentatricopeptide (PPR) repeat-containing protein, a diacylglycerol kinase, a calmodulin-domain protein kinase isoform 9 (CPK9), a family II extracellular lipase 1 (EXL1), two alcohol dehydrogenase 2, an hydroxy-3-methylglutaryl coenzyme A reductase.

In Supplementary Table S2 the DEGs down-regulated in leaves and roots by both EM 0.1 ml L^-1^ and EM 1 ml L^-1^ are also reported. As evinced from the list of DEGs, some of them fall in the same families of genes that are up-regulated by EM, while others are unique for these groups, like early flowering 6 (ELF6), protodermal factor 1 (PDF1), MCM protein, germin like protein, FtsH protease, which are down-regulated in leaves, and phosphatidylinositol 3- and 4-kinase, defense no death 1 (DND1), which are repressed in roots.

### Validation of Gene Expression by qRT-PCR

**FIGURE 7:**
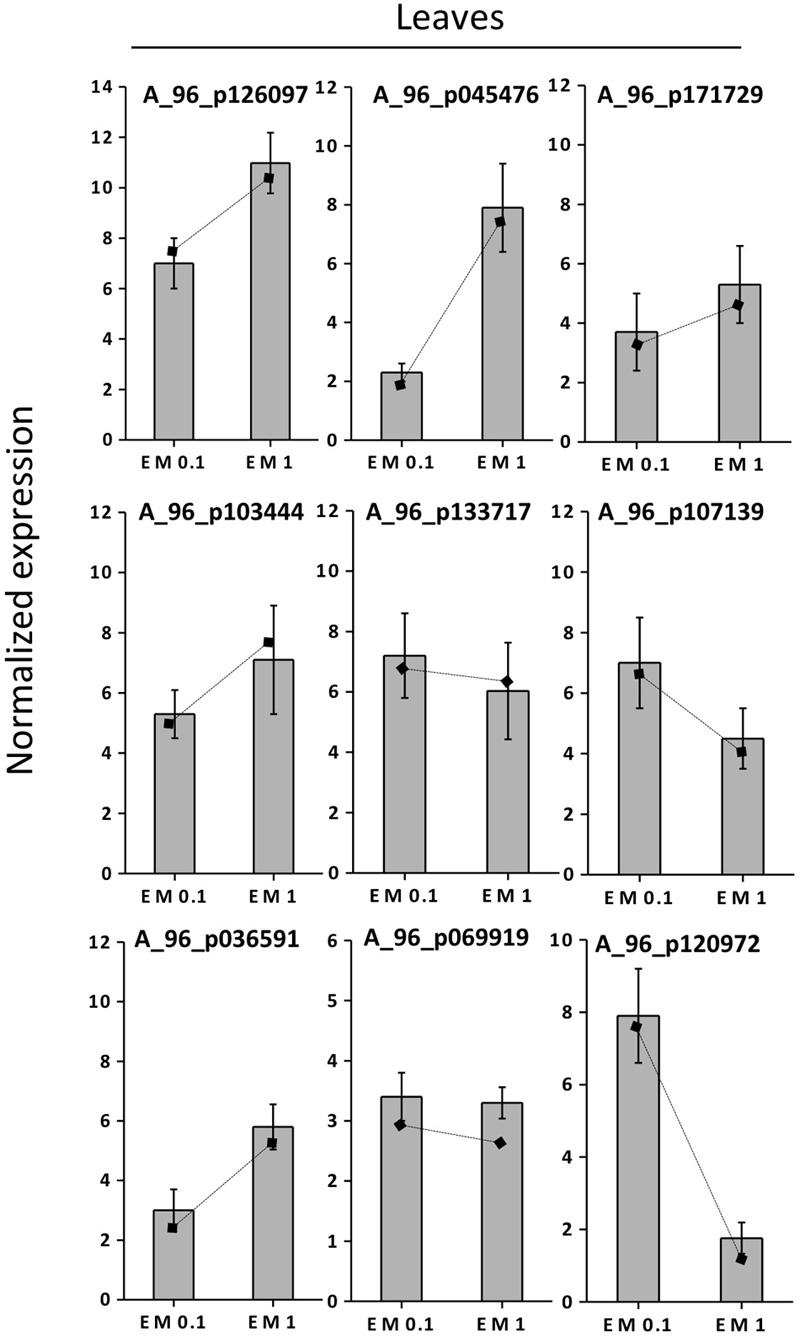
Expression profiling by real-time RT-PCR of selected leaf EM up-regulated genes based on microarray (values are expressed as Log_2_ ratio of normalized intensities). Lines on bars represent values of Log_2_ ratio of normalized intensities from microarray data, and are reported as a comparison with qRT-PCR values. Data shown are the mean ± SD of three replicates.

**FIGURE 8:**
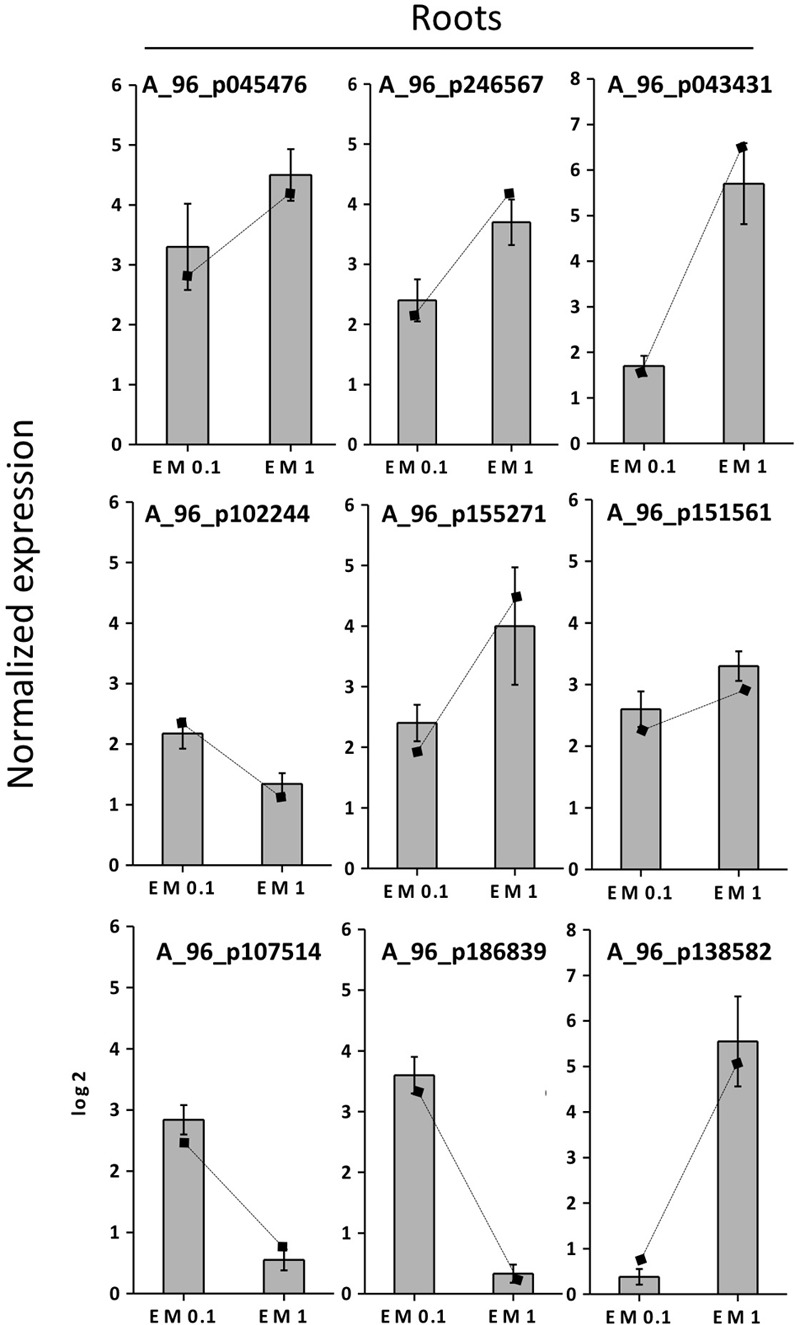
Expression profiling by real-time RT-PCR of selected root EM up-regulated genes based on microarray (values are expressed as Log_2_ ratio of normalized intensities). Lines on bars represent values of Log_2_ ratio of normalized intensities from microarray data, and are reported as a comparison with qRT-PCR values. Data shown are the mean ± SD of three replicates.

To validate the results of the cDNA microarray, 18 EM-induced genes (selected based on cDNA microarray data) were analyzed via qRT-PCR in leaves and roots, using primer pairs with amplification efficiencies ranging within 1.96–1.99. All the 18 genes showed similar expression pattern (*P* < 0.05) in both cDNA microarray and qRT-PCR analysis. Values were expressed as Log_2_ (EM treatment – Control) (**Figures 7, 8**).

The correlation coefficient between the fold-changes data obtained via microarray and those obtained via qRT-PCR for each same expression pattern gene was therefore particularly high being *R*^2^ = 0.97 and 0.89 for leaves and roots, respectively (values calculated on the average data for EM 0.1 ml L^-1^ and EM 1 ml L^-1^, *P* < 0.05). On this account and since gene expression values between microarray and qRT-PCR can vary within 0.48–0.94 and criteria for the determination of an acceptable validation of microarray results by qRT-PCR are hardly definite (Morey et al., 2006), we believe the microarray data of the current study are validated by qRT-PCR experiments.

### Effects of EM on Mineral Content

The product EM was able to promote nutrient accumulation in leaves when applied to plants, regardless of its concentration (**Table 2**). The effect was more pronounced for P and K, as their concentration was about sevenfold higher in EM treated plants than in the untreated. In the case of S and Fe, maximum values were recorded in leaves of plants treated with EM at the concentration of 1 mL L^-1^ (2 and 3.4 higher than the control, respectively). Nitrogen concentration in plants was increased by about 11%.

**Table 2:** Concentration of nitrogen (N), sulfur (S), phosphorus (P), potassium (K), and iron (Fe) in leaves of *Solanum lycopersicon* plants.

	N	S	P	K	Fe
	**% (w/w)**	**mg kg*^-1^***			

Control	5.5 ± 0.1a	1405.2 ± 63.2a	736.2 ± 17.4a	7406.9 ± 46.3a	94.5 ± 12.0a
EM 0.1	6.1 ± 0.2b	2052.2 ± 56.2b	5420.1 ± 223.1b	47716.2 ± 502.2b	124.6 ± 21.2a
EM 1	6.2 ± 0.1b	2840.1 ± 35.1c	4885.7 ± 225.2b	48979.1 ± 439.8b	325.6 ± 23.5b

*Different letters along individual columns indicate significant differences between treatments (*P* < 0.05; *n* = 3, ±SD).*

### Effects of EM on Total Antioxidant Capacity and Content of Phenolic Compounds and Soluble Sugars

The analysis of the TAC in *S. lycopersicon* plants treated with EM at two dosages evidenced the efficacy of the test product to stimulate this parameter (**Table 3**). The degree of TAC improvement by EM 0.1 ml L^-1^ was similar between leaves (+52%) and roots (+58%), and it was generally higher than that caused by EM 1 ml L^-1^ (+115 and +135% in leaves and roots, respectively).

**Table 3:** Content of soluble sugars (glucose and fructose) and total phenols (TP), and total antioxidant capacity (TAC).

	Glucose	Fructose	TP	TAC
	**mg g^-1^ FW**		**mg GA eq kg^-1^ FW**	**mg Fe^2^ kg^-1^ FW**

**Leaves**			
Control	1.96 ± 0.18b	2.45 ± 0.32c	0.36 ± 0.02b	4.63 ± 0.22b
EM 0.1	2.09 ± 0.33b	1.76 ± 0.47b	0.43 ± 0.03a	6.40 ± 0.31a
EM 1	5.61 ± 0.29a	6.41 ± 0.58a	0.41 ± 0.02a	5.15 ± 0.19a
**Roots**				
Control	0.51 ± 0.12	0.32 ± 0.10	0.18 ± 0.02a	1.35 ± 0.12a
EM 0.1	1.22 ± 0.21	1.24 ± 0.15	0.23 ± 0.03a	2.13 ± 0.14b
EM 1	1.11 ± 0.19	0.88 ± 0.17	0.19 ± 0.03a	1.82 ± 0.30b

*Different letters along columns, distinctly for leaves and roots, indicate significant differences (*P* < 0.05) among treatments.*

The same trend was observed for the amount of total phenol compounds (**Table 3**), which was more increased following the application to plants of EM at the lower dose (+26 and +34% in leaves and roots, respectively). Indeed, when EM was applied at 1 ml L^-1^ the total phenol content did not appreciably vary in roots, whereas in leaves it was enhanced by +15% compared to the control plants.

With respect to soluble sugars, the content of these compounds was significantly increased in leaves of plants treated with EM 1 ml L^-1^ (+186 and +161% for glucose and fructose, respectively), but no substantial effect was determined by EM 0.1 ml L^-1^ (**Table 3**). Conversely, EM at both dosages was responsible for higher soluble sugar accumulation in roots. EM at 0.1 ml L^-1^ in particular, caused the maximum increase in glucose (+140%) and fructose (+287%) in this tissue.

## Discussion

The use of biostimulants in agriculture to improve plant yield by enhancing metabolic processes and resistance to abiotic and biotic stresses has attracted growing interest worldwide (Calvo et al., 2014; Nardi et al., 2016; Posmyk and Szafrańska, 2016). These products do not act on plant metabolism directly; rather, they seem to interact with plant-signaling cascades events that trigger the mitigation of negative plant performance responses associated to environmental stress (Brown and Saa, 2015).

Due to the chemical complexity of biostimulant formulation and their content in multiple bioactive substances, the precise molecular mechanisms though which biostimulants act in plants is of hard unraveling (Bulgari et al., 2015; Nardi et al., 2016). On this account, the current study used cDNA microarray in order to get a better framework of the molecular networks that may be envisioned as potential targets for the development of more efficient protein hydrolysates in the market of biostimulants.

Two different concentrations of the biostimulant EM were tested for their effects in tomato, one of the major crop cultivated globally. The in-depth bioinformatic analysis of all the data produced by microarray highlighted that EM induced some important metabolic activities and cell processes in this plant species. Specifically, a number of genes whose expression showed significant variation in response to EM were involved in plant development, photosynthesis, primary C and N metabolism. The up-regulation of these genes justified the greater leaf and root biomass production of tomato plants treated with EM at both dosages compared to the no-treated plants. In a previous work, the increase of growth parameters in tomato plants treated with plant-derived protein hydrolysates was correlated with the stimulation of nitrogen uptake and assimilation (Colla et al., 2014), and in another study the same product EM was reported to promote N assimilation in maize plants via a coordinated up-regulation of the activity of enzymes and expression of genes implied in carbon C and N metabolism (Schiavon et al., 2008). Similarly, in tomato plants we found that EM increased the expression of N assimilation-related genes coding for NR, aspartate AST, glutamine-dependent asparagine synthetase (ASN1) and GS. Additional N-associated genes involved in the synthesis and turnover of amino acids (e.g., glutamate dehydrogenase, serine decarboxylase, aspartyl protease) and in protein synthesis and modification (particularly translation initiation factors, elongation factors Tu and 1-alpha, aminoacyl-tRNA synthetases, ubiquitin-conjugating enzymes, polyubiquitin) were up-regulated by EM. At the same time, we observed higher transcript accumulation of key genes of the major C metabolism, primarily malate dehydrogenase (MDH), phosphoenolpyruvate carboxylase (PEPC), fumarate dehydrogenase (FDH), and phosphoenolpyruvate carboxylase kinase 2 (PPCK2).

Increased N and Fe accumulation and utilization in leaves can account for enhanced photosynthesis and improved translocation of photosynthates from the sources to the sinks that contribute to the improved plant biomass of plants treated with the protein hydrolysate. In support of this hypothesis, EM promoted N and Fe accumulation in tomato plants. In addition, EM increased the expression of a sugar transporter in leaves and SPAD index values, the latters functioning as indicators of chlorophyll production and photosynthetic efficiency. It also induced the up-regulation of genes coding for components of the photosynthetic electron transfer chain (e.g., ferredoxin-2, the light-harvesting complex protein LHCA5, a chloroplast ATP synthase chain precursor) and the enzyme Ribulose-1,5-bisphosphate carboxylase/oxygenase (RuBisCo), responsible for the process of CO_2_ fixation in the Calvin cycle. As a result of this positive effect on photosynthesis, the increase in content of soluble sugars (glucose and fructose) was observed in EM-treated tomato plants. Previous studies reported higher accumulation of sugars (Schiavon et al., 2008; Ertani et al., 2011a) and RuBisCo activity (Ertani et al., 2011a) in maize plants after application of either EM or lignosulfonate-humates.

It is noteworthy that EM in tomato plants stimulated the transcription of a gene coding for a photosystem II 22 kda protein, which is not strictly necessary for efficient light harvesting and photosynthesis, but plays a key role in non-photochemical quenching, a process that preserves the balance between dissipation and utilization of light energy to minimize generation of reactive oxygen species (ROS), thereby preventing plants from photo-oxidative damages.

In addition to improved nutrient use efficiency and photosynthesis, EM influenced the capacity of tomato plants to absorb, translocate and allocate nutrients in different organs by modulating the expression of genes coding for ATPases and proteins that mediate the transport of inorganic elements (e.g., N, S, P, K, Cu, Fe) and organic molecules (mainly amino acids, peptides and sugars) over cells membranes.

EM treatment, for instance, induced higher expression of the nitrate transporter NTP2, which is homolog to the *Arabidopsis thaliana* AtNRT1;4 functioning in leaf nitrate homeostasis (Chiu et al., 2004), and the ammonium transporter AMT1.1. Genes coding for sulfate transporters like SULTR 2;1, which plays a role in xylem loading and root-to-shoot transport of sulfate (Kawashima et al., 2011; Maruyama-Nakashita et al., 2015), and SULTR 3;1, which is chloroplast localized and mediates the entry of sulfate into the plastids for assimilation into S-amino acids (Cao et al., 2013), as well as the phosphate transporter PT2, the iron-phytosiderophore transporter protein yellow stripe 1 (YS1), potassium channels and copper transporters, were also increased in expression by EM. The up-regulation of nitrate and sulfate transporters, as well as ATPases, by biostimulants was previously described in other plant species (Canellas et al., 2002; Quaggiotti et al., 2004; Jannin et al., 2013). These data indicate that EM can promote the transport of nutrients in tomato plants likely by acting on cell membranes properties.

Consistently with higher expression of nutrient transporters, EM treated-tomato plants exhibited higher foliar accumulation of mineral elements such as N, S, P, K, and Fe, with the most pronounced increase reported for P and K. The effect on mineral nutrition was also reported in a recent study conducted in maize plants using another protein-based hydrolysate (Santi et al., 2017).

The way through which EM modifies the membrane permeability to favor the movement of nutrients may be at least partly ascribed to changes in root architecture shape and development via an auxin-signaling mediated pathway. The EM characterization described in Ertani et al. (2014) reported the presence of IAA (18.5 nmol mg^-1^ C) in the formulation. Previous work showed that auxin-like molecules of microbial origin contained in humic substances could influence plant growth by eliciting auxin-dependent signals that enhance the production of lateral root formation (Trevisan et al., 2010). Several genes encoding for auxin-responsive proteins had significant higher expression in EM-treated tomato plants and might be partly responsible for the observed increase in root biomass. Furthermore, a number of growth regulating factors (GRF3, GRF5), lob domain proteins, and expansins genes were up-regulated by EM. Expansins particularly, mediate cell wall loosening during cell growth and may have a role in improving stress tolerance (Marowa et al., 2016). Expression of expansins was correlated with expression of genes involved in the synthesis (e.g., cellulose synthase) and degradation (e.g., pectinesterases, polygalacturonases, and lipases) of cell wall and membrane structural components.

There are evidences that biostimulants help plants to overcome different biotic and abiotic stress situations (Joubert and Lefranc, 2008; Ertani et al., 2013b). Several EM-responsive genes identified via microarray were implied in detoxification and oxidative stress resistance. Tomato plants treated with EM showed increased TAC of ROS that are usually generated at high levels under stress. Among the genes with a key role in mitigating oxidative stress, the main represented were glutathione peroxidase (GPX), glutathione reductase (GR), GST, peroxidases, thioredoxins, and DHAR. Interestingly, most of these genes are implied in the glutathione/ascorbate detoxifying cycle, thereby suggesting that this pathway may be an important target of the biostimulant mode of action.

Also, EM treatment caused the up-regulation of genes involved in defense systems and plant-organism interactions, perhaps via modulation of the synthesis and signaling of defense hormones [ethylene, jasmonic acid, abscissic acid (ABA), salicylic acid (SA)] by elicitors (phytoactivators) contained in the biostimulant, such as auxins, phenols, amino acids and peptides. Algae extracts from *Laminaria digitata*, for instance, can induce natural immunity/resistance in plants without exerting any direct antimicrobial activity by virtue of their content in phytoactivators (Joubert and Lefranc, 2008). In support of our hypothesis, the expression of ethylene biosynthetic genes and ethylene/JA/ABA- responsive genes was higher in plants endowed with EM. The hormone signaling pathway elicited by EM likely triggers a cascade of phosphorylation events mediated by a variety of protein kinases (primarily MAPKKK21, CPK28, CRCK3, Pi kinase, LRR kinases, CPK9, WAK2, PEPKR2), which ultimately leads to the transcription of defense-related genes (particularly Cytochrome 450, leucine rich repeat proteins, heat shock proteins, aldo/keto reductase, glutathione-*S*-transferases, threonine ammonia-lyase and chitinases, lactoylglutathione lyase, DNAJ, pathogenesis-related protein Bet v, subtilases, DREB2A, hydroxyproline-rich glycoprotein, wound-induced proteins, L-threonine ammonia-lyase, alternative oxidase 1A, syntaxin, DC1 domain-containing proteins, thaumatin, ECERIFERUM, ABC transporters). Some of these genes are mainly involved in abiotic stress, like heat shock proteins and wound-induced proteins, but most of them are important in plant defense against pathogens or herbivores.

A special mention is for ABC transporters, initially identified as transporters involved in detoxification processes, later recognized as crucial for organ growth, plant nutrition, plant development, response to abiotic stresses, pathogen resistance, hormone transport, and interaction of the plant with its environment (Kang et al., 2011). Their up-regulation indicates that a crosstalk of signaling events occurs in plants in response to EM application, which regulates plant primary metabolism, development and defense in plants.

Important part of this crosstalk are EM-responsive genes involved in the secondary metabolism. The hormone ethylene, whose synthesis seems to be stimulated by EM, can positively influence N assimilation and the secondary metabolism associated with the synthesis of phenols and terpenes (Khan et al., 2015). These compounds serve a dual function of deterring invading organisms and attracting pollinators (Lattanzio et al., 2006; Vermerris and Nicholson, 2006; Chen et al., 2011; Zhang et al., 2016). Phenols are also critical for plant development, especially in the synthesis of lignin and pigments, while terpenes include carotenoids, which are important for light harvesting and protection from excess of light radiation. Among the genes identified in tomato plants within this category, the one coding for PAL is of particular interest. PAL is a key enzyme of the phenol biosynthetic pathway and its activity and gene expression was previously shown to increase in response to applications of different biostimulants (Schiavon et al., 2010; Ertani et al., 2011a) and high ethylene levels (Chalutz, 1973). The increase in expression of this gene in tomato plants treated with EM correlated with higher production of phenol compounds they display, thereby providing strong evidence that phenol metabolism is a major target of biostimulants in this species.

Many transcription factors showed variation in expression in response to EM, but the most dramatic change was detected for a basic helix-loop-helix (bHLH) protein, which is known to play a key role in a multiplicity of transcriptional programs related to abiotic stress and plant development (Xu et al., 2014). Among the most largely represented EM-responsive transcription factors identified were AP2/ EREBPs (APETALA2) and WRKY, both involved in abiotic and biotic stress responses and in developmental processes, zinc finger (ZFN) proteins regulating development, growth, stress responses and phytohormone responses (Sakamoto et al., 2004), pentatricopeptide repeat-containing protein (PPR), which facilitate processing, splicing, editing, stability and translation of RNAs (Manna, 2015), bzip proteins, which have a critical role in photomorphogenesis, leaf and seed formation, energy homeostasis, and abiotic and biotic stress responses (Corrêa et al., 2008).

Comparing the effects of different EM concentrations on plant performance and gene expression in tomato, despite EM at 1 mL L^-1^ influenced plant growth, SPAD index and sugars more positively than EM at 0.1 mL L^-1^, we can conclude that both dosages elicited the main metabolic pathways previously described, thereby suggesting that our data and hypothesized mechanisms for EM mode of action are fairly consistent.

## Conclusion

**FIGURE 9:**
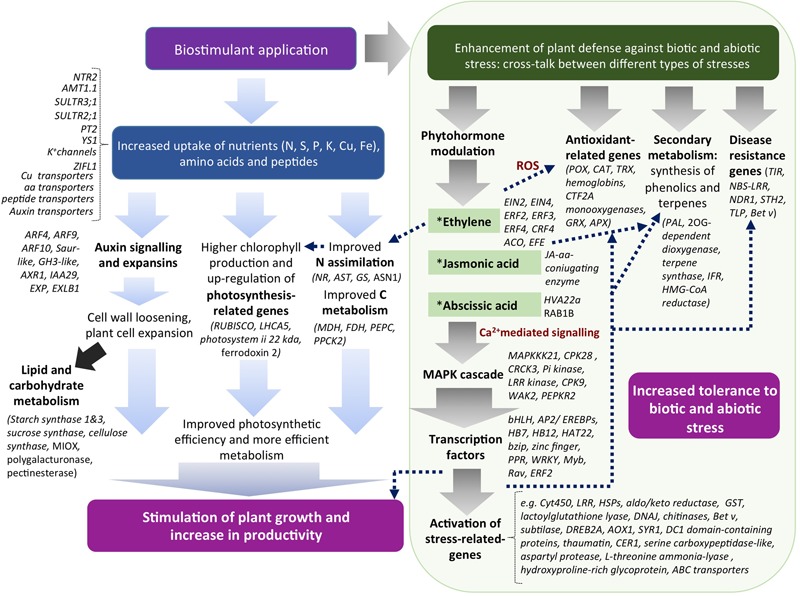
Model for EM biostimulant mode of action in plants and their correlations. EM can stimulate plant productivity via up-regulation of genes related to N and C metabolism, photosynthesis, development and auxin-related. At the same time, EM by virtue of phytoactivators contained in its formulation (e.g., auxins, phenols, and amino acids) can modulate the level of stress-related hormones, thus activating a cascade of events that ultimately causes the up-regulation of both genes involved in defense, antioxidant activities, plant interactions with organisms, growth and development.

The alfalfa-based protein hydrolysate tested in this study showed effectiveness as a biostimulant in tomato plants by enhancing plant productivity via multidirectional mechanisms. Some metabolic pathways can be definitely recognized as targets of EM action in plants, such as N and C primary metabolism, photosynthesis, transport of nutrients, secondary metabolism associated with the synthesis of phenolic and terpene compounds, and developmental processes related to auxin signaling. Furthermore, a number of new genes have been identified in tomato as potential targets of EM, such as those involved in plant-organism interactions, detoxification (glutathione/ascorbate cycle-related, ABC transporters), and defense against abiotic stress. In **Figure 9** a hypothetic model that represents a possible mode of action of EM in plants is depicted.

We conclude that the EM can act as a biostimulant in tomato plants may improve plant productivity and eliciting resistance responses, thereby reducing the need of conventional treatments that employ inorganic fertilizers and pesticides in agricultural practices and impact on the environment.

## Author Contributions

AE performed the experiments and wrote the ms. MS analyzed the data and wrote the ms. SN supervised the work and provided financial support.

## Conflict of Interest Statement

The authors declare that the research was conducted in the absence of any commercial or financial relationships that could be construed as a potential conflict of interest.

## Footnotes

1 http://gabi.rzpd.de/projects/MapMan/

2 http://www.bioinformatics.lu/venn.php
