# Supplementary material for: Transcriptome-Wide Identification of Differentially Expressed Genes in Solanum lycopersicon L. in Response to an Alfalfa-Protein Hydrolysate Using Microarrays
Source: Front Plant Sci. 2017 Jul 5;8:1159. doi: 10.3389/fpls.2017.01159 (PMC5496959; doi:10.3389/fpls.2017.01159)
Supplement: Supplementary file 1 [file Data_Sheet_1.docx]

**Supplementary Material.**

**Table 1S. List of primers used in qRT-PCR reactions for validation of microarray data.**

| **Agilent gene ID** | ***Forward 5’-3’*** | | ***Reverse 5’-3’*** |  |
| --- | --- | --- | --- | --- |
| **A_96_p126097** | TAGCGATTTACAGGGAGTGG | | AGCAAAGAAGCGGAAGGTTT |  |
| **A_96_p045476** | TCCATAAGCAAATCCCAGTT | | TGTCCATACCAATCTCCCTGT |  |
| **A_96_p171729** | AGACGATACGCTCCCTGATG | | TTTACCCCAAATACCCTCCA |  |
| **A_96_p103444** | ACGGATTGTAAGGCAACCTG | | CCCTCAACGGAGCATCAC |  |
| **A_96_p133717** | GACGCTGACTCCACAAACG | | GCTTCTTCTTCGCCATTGAA |  |
| **A_96_p107139** | TCCAGGGGAAGGATTCAAC | | CTTTGTTTAGGGTCGGGTCA |  |
| **A_96_p035591** | CAGTTGAGCGGGTAAGTAGGA | | CGAGGAGGTTGAGCAGAAAC |  |
| **A_96_p069919** | GCAAATGGTGGCAAAATAAC | | AGCACAAGATGAGCAAGAACC |  |
| **A_96_p120972** | CCCACACAGAACTCAAGCAA | | GCCTTTCCTCCCGTAAAATC |  |
| **A_96_p045476** | TCCATAAGCAAATCCCAGTT | | TGTCCATACCAATCTCCCTGT |  |
| **A_96_p246567** | GACAAAAGAGTTGCCCACAGA | | AGTTTCCATTCTCGCCACCT |  |
| **A_96_p043431** | | GAAACTGGTGCTTGTGGATT | TCTGATGCTGCCGATACAT | |
| **A_96_p102244** | CGAGGGGACCAGAATAGGAG | | TAAGGCTCTACCAGGCGAAA |  |
| **A_96_p155271** | TGACAGGGGAGTGATGCTTA | | AAAATGGTAAATGGGGTTTCC |  |
| **A_96_p151561** | GGCTGATGTGGATGGAAATG | | AATGGACTTACCCGCTACCA |  |
| **A_96_p107514** | GTTGGTCGGTGATTGTTCCT | | GGATTTCGGATAAACGGGTAA |  |
| **A_96_p186839** | TTCAGCCCAAAGAGCAGTG | | CGACCCGTAATGGACACAT |  |
| **A_96_p138582** | CGGAGTTCAAGCCAGAGAG | | GCCATTTCTTTCCCTAAGCA |  |
