# Supplementary material for: Transcriptome-Wide Identification of Differentially Expressed Genes in Solanum lycopersicon L. in Response to an Alfalfa-Protein Hydrolysate Using Microarrays
Source: Front Plant Sci. 2017 Jul 5;8:1159. doi: 10.3389/fpls.2017.01159 (PMC5496959; doi:10.3389/fpls.2017.01159)
Supplement: Supplementary file 2 [file Data_Sheet_2.docx]

**Table 2S. List of gene sequences regulated in leaves and roots of tomato plants treated with either EM 0.1 mL L^-1^ or EM 1 mL L^-1^. Annotation is given based on blastx search against the plant sequence database. On the left of the table, the Agilent probe ID is reported.**

| **Leaves of tomato plants treated with either EM 0.1 or EM 1 mL L^-1^: *up-regulation by both treatments*** | | | | |
| --- | --- | --- | --- | --- |
| **Agilent ID** | | **Fold change** | | **Annotation** |
|  |  | *EM 0.1 mL L^-1^* | *EM 1 mL L^-1^* |  |
| A_96_p132312 | | 1170.57 | 2.43 | basic helix-loop-helix (bHLH) |
| A_96_p011406 | | 265.40 | 265.31 | aldo/keto reductase |
| A_96_p126097 | | 177.42 | 1170.18 | expansin |
| A_96_p120972 | | 176.80 | 2.63 | phox (PX) domain-containing protein |
| A_96_p133717 | | 126.68 | 85.78 | AP2 domain-containing transcription factor, putative |
| A_96_p107139 | | 99.59 | 15.92 | peroxidase, putative |
| A_96_p103444 | | 28.46 | 115.49 | transferase |
| A_96_p119612 | | 16.83 | 2.46 | homeobox-leucine zipper |
| A_96_p131332 | | 16.60 | 2.58 | putative bzip transcription factor protein |
| A_96_p143491 | | 13.75 | 2.58 | lactoylglutathione lyase |
| A_96_p171729 | | 11.17 | 27.52 | Cytochrome P450 94A1 (CYP94C1) |
| A_96_p156756 | | 10.51 | 2.98 | transferase |
| A_96_p069919 | | 7.37 | 6.24 | FED A (FERREDOXIN 2) |
| A_96_p036591 | | 6.43 | 33.07 | homeobox-leucine zipper protein 12 (HB-12) |
| A_96_p079089 | | 6.11 | 6.30 | Protein phosphatase 2C (PP2C) |
| A_96_p052716 | | 6.00 | 3.03 | hydroxyproline-rich glycoprotein |
| A_96_p166274 | | 5.98 | 8.77 | homeobox-leucine zipper protein 7 (HB-7) |
| A_96_p154596 | | 5.74 | 2.51 | 1-aminocyclopropane-1-carboxylate oxidase homolog |
| A_96_p248727 | | 5.67 | 0.43 | copper-exporting ATPase, putative |
| A_96_p091004 | | 5.32 | 5.87 | zinc finger (C3HC4-type RING finger) |
| A_96_p149636 | | 5.14 | 5.70 | protein phosphatase 2C, putative |
| A_96_p116237 | | 5.07 | 6.38 | DNAJ heat shock N-terminal domain-containing protein |
| A_96_p131422 | | 5.05 | 6.30 | OTU-like cysteine protease |
| A_96_p045946 | | 4.90 | 5.99 | Wound-induced protein 1 |
| A_96_p232959 | | 4.84 | 3.33 | ribonuclease 2 (RNS2) |
| A_96_p156186 | | 4.80 | 2.46 | phosphosulfolactate synthase-related protein |
| A_96_p127927 | | 4.68 | 10.69 | zinc finger (Ran-binding) |
| A_96_p171334 | | 4.51 | 18.34 | L-threonine ammonia-lyase |
| A_96_p061761 | | 4.39 | 2.42 | zinc finger (B-box type) |
| A_96_p126452 | | 4.37 | 3.02 | UBC28; ubiquitin-protein ligase |
| A_96_p199644 | | 4.30 | 4.38 | formate dehydrogenase, mitochondrial precursor |
| A_96_p210739 | | 4.23 | 3.43 | jasmonic acid-amino acid-conjugating enzyme |
| A_96_p086379 | | 4.20 | 3.19 | DC1 domain-containing protein |
| A_96_p176349 | | 4.19 | 3.07 | S-locus lectin protein kinase |
| A_96_p126782 | | 4.17 | 2.79 | aldo/keto reductase |
| A_96_p045476 | | 4.07 | 17.40 | glutathione S-transferase |
| A_96_p237397 | | 4.05 | 2.80 | heat shock transcription factor |
| A_96_p178034 | | 4.01 | 2.65 | 2-oxoglutarate-dependent dioxygenase, putative |
| A_96_p096214 | | 3.94 | 2.44 | zinc finger (CCCH-type) |
| A_96_p074534 | | 3.89 | 2.46 | UGT73B3 (UDP-GLUCOSYL TRANSFERASE 73B3) |
| A_96_p093284 | | 3.86 | 4.25 | phosphoenolpyruvate carboxylase |
| A_96_p176374 | | 3.84 | 4.47 | DNA-binding protein |
| A_96_p006481 | | 3.66 | 3.49 | 2OG-Fe(II) oxygenase |
| A_96_p178173 | | 3.51 | 2.65 | 2-oxoglutarate-dependent dioxygenase, putative |
| A_96_p253190 | | 3.49 | 2.46 | WRKY transcription factor |
| A_96_p124337 | | 3.45 | 16.59 | CTF2A monooxygenase |
| A_96_p102219 | | 3.44 | 2.84 | 2OG-Fe(II) oxygenase |
| A_96_p008426 | | 3.43 | 3.55 | universal stress protein (USP) |
| A_96_p033111 | | 3.29 | 13.04 | CTF2A monooxygenase |
| A_96_p048256 | | 3.27 | 0.35 | peroxidase |
| A_96_p067861 | | 3.21 | 7.96 | phosphatidylethanolamine binding protein |
| A_96_p152261 | | 3.16 | 2.03 | zinc finger (C3HC4-type RING finger) |
| A_96_p054931 | | 3.14 | 5.74 | TAF11 (TBP-associated factor 11) |
| A_96_p110017 | | 3.05 | 3.14 | UDP-GLUCOSYLTRANSFERASE 74F2) |
| A_96_p091104 | | 3.11 | 5.23 | NAP (NAC-LIKE, ACTIVATED BY AP3/PI) |
| A_96_p043686 | | 3.09 | 10.68 | hydrolase |
| A_96_p018876 | | 3.08 | 2.84 | IAA29 (indoleacetic acid-induced protein 29) |
| A_96_p014241 | | 3.08 | 126.69 | AOX1A (alternative oxidase 1A) |
| A_96_p110017 | | 3.05 | 3.14 | UDP-GLUCOSYLTRANSFERASE 74F2 |
| A_96_p096049 | | 2.97 | 5.79 | copper amine oxidase, putative |
| A_96_p124242 | | 2.93 | 2.53 | monovalent cation:proton antiporter |
| A_96_p145331 | | 2.85 | 2.19 | Cytochrome P450 71B14, putative (CYP71B14) |
| A_96_P029841 | | 2.84 | 2.39 | phytochelatin synthetase, putative |
| A_96_p110537 | | 2.78 | 2.69 | SRG1 (SENESCENCE-RELATED GENE 1) |
| A_96_p055721 | | 2.77 | 5.23 | homeobox-leucine zipper protein 12 (HB-12) |
| A_96_p194619 | | 2.73 | 2.78 | glutathione S-transferase |
| A_96_p161881 | | 2.72 | 2.36 | UDP-glucoronosyl/UDP-glucosyl transferase |
| A_96_p091119 | | 2.69 | 5.34 | acetolactate synthase |
| A_96_p095329 | | 2.64 | 2.08 | SBP1 (S-RIBONUCLEASE BINDING PROTEIN 1) |
| A_96_p201145 | | 2.63 | 2.31 | FMO1 (FLAVIN-DEPENDENT MONOOXYGENASE 1) |
| A_96_p074234 | | 2.63 | 8.76 | UDP-glucoronosyl/UDP-glucosyl transferase |
| A_96_p118122 | | 2.63 | 4.58 | cinnamoyl-CoA reductase-related |
| A_96_p060146 | | 2.62 | 3.19 | glutaredoxin |
| A_96_p005886 | | 2.61 | 5.27 | In2-1 protein, putative |
| A_96_p174554 | | 2.48 | 2.66 | 2-oxoglutarate-dependent dioxygenase, putative |
| A_96_p038221 | | 2.40 | 5.38 | AP2 domain-containing transcription factor, putative |
| A_96_p073889 | | 2.37 | 2.60 | ERF2 (ETHYLENE RESPONSE FACTOR 2) |
| A_96_p068711 | | 2.17 | 2.45 | scarecrow transcription factor |
| A_96_p210029 | | 2.17 | 2.51 | heat shock protein 101 |
| **Leaves of tomato plants treated with either EM 0.1 or EM 1 mL L^-1^: *down-regulation in both treatments*** | | | | |
| **Agilent ID** | | **Fold change** | | **Annotation** |
|  | | *EM 0.1 mL L^-1^* | *EM 1 mL L^-1^* |  |
| A_96_p201884 | | 0.03 | 0.43 | purple acid phosphatase |
| A_96_p110637 | | 0.09 | 0.33 | FAD-binding domain-containing protein |
| A_96_p168864 | | 0.11 | 0.41 | purple acid phosphatase |
| A_96_p226109 | | 0.11 | 0.20 | very-long-chain fatty acid condensing enzyme, putative |
| A_96_p232954 | | 0.13 | 0.41 | ELF6 (EARLY FLOWERING 6) |
| A_96_p073034 | | 0.15 | 0.40 | WRKY33 (WRKY DNA-binding protein 33) |
| A_96_p225819 | | 0.16 | 0.14 | aldo/keto reductase, putative |
| A_96_p121462 | | 0.17 | 0.40 | MATE efflux |
| A_96_p186619 | | 0.18 | 0.38 | Glutaredoxin (thioltransferase) |
| A_96_p019296 | | 0.20 | 0.31 | ribosomal protein L11 methyltransferase-related |
| A_96_p221829 | | 0.20 | 0.41 | chloride channel-like (CLC) protein, putative |
| A_96_p064461 | | 0.20 | 0.31 | minichromosome maintenance |
| A_96_p169579 | | 0.21 | 0.19 | protodermal factor 1 (PDF1) |
| A_96_p138712 | | 0.23 | 0.28 | cytoplasmic small heat shock protein class |
| A_96_p015511 | | 0.24 | 0.49 | oxidoreductase |
| A_96_p195884 | | 0.25 | 0.43 | leucine-rich repeat transmembrane protein kinase, putative |
| A_96_p218349 | | 0.26 | 0.32 | GTP-binding protein-related |
| A_96_p123472 | | 0.26 | 0.42 | kinesin motor protein-related |
| A_96_p146486 | | 0.26 | 0.31 | ZIFL1 (ZINC INDUCED FACILITATOR-LIKE 1 |
| A_96_p047306 | | 0.28 | 0.21 | pectinesterase |
| A_96_p203904 | | 0.29 | 0.37 | germin like protein |
| A_96_p064286 | | 0.29 | 0.31 | alpha-amylase |
| A_96_p227009 | | 0.30 | 0.30 | cytokinin dehydrogenase |
| A_96_p225814 | | 0.31 | 0.17 | UBQ6 (ubiquitin 6) |
| A_96_p017641 | | 0.31 | 0.26 | ICE1 (INDUCER OF CBF EXPRESSION 1) |
| A_96_p146056 | | 0.31 | 0.30 | PLP4 (Patatin-like protein 4) |
| A_96_p107934 | | 0.31 | 0.38 | kinase |
| A_96_p186394 | | 0.31 | 0.41 | alpha-amylase |
| A_96_p116462 | | 0.32 | 0.22 | histidine kinase |
| A_96_p060111 | | 0.32 | 0.41 | EAD/DEAH box helicase, putative (RH22) |
| A_96_p204919 | | 0.32 | 0.21 | CMT3 (CHROMOMETHYLASE 3) |
| A_96_p216314 | | 0.33 | 0.38 | leucine-rich repeat transmembrane protein kinase, putative |
| A_96_p208944 | | 0.33 | 0.41 | glutamyl-tRNA(Gln) amidotransferase, putative |
| A_96_p135067 | | 0.35 | 0.26 | ribosomal protein L13 |
| A_96_p045641 | | 0.35 | 0.39 | mitochondrial substrate carrier |
| A_96_p002456 | | 0.35 | 0.24 | TUA6 (tubulin alpha-6 chain) |
| A_96_p207924 | | 0.35 | 0.41 | gtp-binding-like protein |
| A_96_p021691 | | 0.37 | 0.32 | GDSL-motif lipase/hydrolase |
| A_96_p203324 | | 0.38 | 0.41 | leucine-rich repeat transmembrane protein kinase, putative |
| A_96_p051926 | | 0.38 | 0.30 | MCM protein-like protein |
| A_96_p053536 | | 0.40 | 0.43 | pfkB-type carbohydrate kinase |
| A_96_p133867 | | 0.40 | 0.29 | photoassimilate-responsive protein-related |
| A_96_p181439 | | 0.40 | 0.27 | calcium-transporting ATPase, plasma membrane-type, putative |
| A_96_p053251 | | 0.41 | 0.43 | acid phosphatase |
| A_96_p205724 | | 0.41 | 0.30 | GDSL-motif lipase/hydrolase |
| A_96_p032771 | | 0.41 | 0.42 | kinase |
| A_96_p022976 | | 0.41 | 0.28 | zinc finger (CCCH-type) |
| A_96_p232219 | | 0.41 | 0.26 | PHD finger |
| A_96_p165776 | | 0.41 | 0.34 | glucose-methanol-choline (GMC) oxidoreductase |
| A_96_p123652 | | 0.42 | 0.28 | protein kinase |
| A_96_p177944 | | 0.42 | 0.33 | GDSL-motif lipase/hydrolase |
| A_96_p149746 | | 0.42 | 0.32 | germin like protein |
| A_96_p023396 | | 0.42 | 0.43 | FtsH protease |
| A_96_p125452 | | 0.42 | 0.30 | cysteine proteinase, putative |
| A_96_p046976 | | 0.43 | 0.43 | Heavy metal transport/detoxification protein |
| A_96_p100054 | | 0.43 | 0.43 | CAC2 (acetyl co-enzyme A carboxylase biotin carboxylase subunit) |
| A_96_p110527 | | 0.43 | 0.24 | kinesin motor protein-related |
| Leaves of tomato plants treated with either EM 0.1 mL L^-1^ or EM 1 mL L^-1^: ***up-regulation in EM0.1 and down-regulation or no effect in EM1*** | | | | |
| **Agilent ID** | | **Fold change** | | **Annotation** |
|  | | *EM 0.1 mL L^-1^* | *EM 1 mL L^-1^* |  |
| A_96_p177249 | | 625.99 | 0.98 | polypyrimidine tract-binding protein, putative |
| A_96_p218799 | | 321.80 | 1.24 | EMB1379 (EMBRYO DEFECTIVE 1379) |
| A_96_p142287 | | 142.12 | 1.56 | EMB1303 (EMBRYO DEFECTIVE 1303) |
| A_96_p193959 | | 36.30 | 1.12 | lipase class 3 |
| A_96_p150911 | | 33.34 | 0.96 | non-symbiotic hemoglobin class 1 |
| A_96_p253832 | | 31.89 | 0.89 | RNA recognition motif (RRM)-containing protein |
| A_96_p165476 | | 21.80 | 1.08 | inositol oxygenase |
| A_96_p066476 | | 21.20 | 1.10 | cellulose synthase catalytic subunit |
| A_96_p239058 | | 19.92 | 1.21 | nodulin MtN21 |
| A_96_p117017 | | 15.26 | 1.24 | zinc finger (CCCH-type) |
| A_96_p093720 | | 14.53 | 1.04 | peroxidase, putative |
| A_96_p147006 | | 13.36 | 0.96 | peroxidase, putative |
| A_96_p103619 | | 13.03 | 0.98 | ribose-phosphate pyrophosphokinase 2 |
| A_96_p085154 | | 12.95 | 0.99 | putative kiwellin ripening-related protein precursor |
| A_96_p176125 | | 11.27 | 1.58 | peroxidase, putative |
| A_96_p203499 | | 10.99 | 0.32 | inositol oxygenase |
| A_96_p216104 | | 10.68 | 1.76 | MATE efflux |
| A_96_p046226 | | 9.17 | 1.54 | cytochrome P450 82C2 (CYP82C2) |
| A_96_p032486 | | 8.53 | 1.05 | protease inhibitor |
| A_96_p233539 | | 8.35 | 1.14 | delta tonoplast intrinsic protein TIP2;3 |
| A_96_p210664 | | 7.96 | 0.92 | peroxidase, putative |
| A_96_p095609 | | 7.92 | 1.08 | translation initiation factor |
| A_96_p151561 | | 7.73 | 1.52 | calcium-dependent protein kinase 33 |
| A_96_p178454 | | 7.16 | 1.10 | pollen Ole e 1 allergen and extensin |
| A_96_p105109 | | 6.99 | 0.89 | peroxidase, putative |
| A_96_p151351 | | 6.83 | 0.45 | F-box |
| A_96_p210459 | | 6.66 | 1.06 | cell division cycle protein 48, putative |
| A_96_p059761 | | 6.48 | 1.22 | PIL5 (PHYTOCHROME INTERACTING FACTOR 3-LIKE 5) |
| A_96_p141092 | | 6.48 | 1.28 | gibberellin 2-beta-dioxygenase |
| A_96_p236093 | | 6.41 | 1.08 | lactoylglutathione lyase |
| A_96_p176699 | | 6.24 | 1.43 | ATP binding protein |
| A_96_p209824 | | 5.79 | 1.51 | cupin |
| A_96_p107524 | | 5.68 | 1.91 | Cytochrome P450 (CYP71A22) |
| A_96_p031861 | | 5.63 | 1.03 | putative dnaj protein |
| A_96_p192329 | | 5.62 | 0.87 | homeobox-leucine zipper |
| A_96_p184239 | | 5.43 | 0.99 | chitinase |
| A_96_p026358 | | 5.42 | 1.20 | peroxidase, putative |
| A_96_p189919 | | 5.29 | 1.67 | dehydroascorbate reductase |
| A_96_p146536 | | 5.23 | 1.64 | 2-oxoglutarate-dependent dioxygenase, putative |
| A_96_p058376 | | 5.11 | 1.32 | aminoacyl-tRNA synthetase |
| A_96_p197854 | | 5.03 | 1.05 | protein kinase |
| A_96_p108292 | | 4.99 | 1.66 | protein phosphatase 2C |
| A_96_p187999 | | 4.88 | 0.87 | lysine and histidine specific transporter, putative |
| A_96_p188164 | | 4.86 | 0.45 | AMP deaminase, putative |
| A_96_p232979 | | 4.85 | 1.09 | ABA-responsive protein-related |
| A_96_p124542 | | 4.83 | 1.79 | phosphoenolpyruvate carboxykinase1 |
| A_96_p087284 | | 4.78 | 1.92 | peptide-methionine-(S)-S-oxide reductase |
| A_96_p120962 | | 4.77 | 1.68 | amino acid permease |
| A_96_p054486 | | 4.75 | 1.06 | wound-responsive |
| A_96_p052346 | | 4.66 | 0.94 | aspartate aminotransferase 2 |
| A_96_p211504 | | 4.64 | 1.36 | UDP-glycosyltransferase |
| A_96_p140762 | | 4.56 | 1.16 | leucine-rich repeat |
| A_96_p030906 | | 4.47 | 1.53 | auxin-responsive protein, putative |
| A_96_p184249 | | 4.44 | 1.07 | CCAAT-binding transcription factor (CBF-B/NF-YA) |
| A_96_p168814 | | 4.40 | 0.99 | polyprotein, putative |
| A_96_p214529 | | 4.32 | 1.68 | mads box protein |
| A_96_p010156 | | 4.30 | 1.54 | Glutathione S-transferase, predicted |
| A_96_p043431 | | 4.29 | 1.30 | expansin EXLB1 |
| A_96_p095924 | | 4.28 | 1.23 | pectate lyase |
| A_96_p231499 | | 4.25 | 1.09 | glycoside hydrolase family 2 protein |
| A_96_p102244 | | 4.20 | 0.93 | Multidrug resistance-associated protein |
| A_96_p155026 | | 4.17 | 1.45 | armadillo/beta-catenin repeat |
| A_96_p169764 | | 4.13 | 0.84 | auxin influx transport protein |
| A_96_p172444 | | 4.11 | 0.91 | myosin heavy chain (MYA2) |
| A_96_p146881 | | 4.11 | 1.67 | calmodulin-dependent protein kinase/ kinase |
| A_96_p107309 | | 4.06 | 1.55 | leucine-rich repeat transmembrane protein kinase, putative |
| A_96_p145661 | | 4.04 | 1.34 | ein3-binding f-box protein 1 |
| A_96_p113942 | | 4.04 | 1.07 | putative f-box protein |
| A_96_p181024 | | 4.04 | 1.10 | Heat shock protein 91 |
| A_96_p228994 | | 4.02 | 0.56 | acyl-activating enzyme 11 (AAE11) |
| A_96_p093284 | | 4.02 | 1.08 | wound induced protein |
| A_96_p093284 | | 3.83 | 1.19 | Phosphoenolpyruvate carboxylase |
| A_96_p040166 | | 3.73 | 1.50 | Cytochrome P450 714A1 (CYP714A1) |
| A_96_p184134 | | 3.66 | 0.60 | Cytochrome P450 71A22 CYP71A22) |
| A_96_p179189 | | 3.56 | 1.12 | Dof-type zinc finger domain-containing protein |
| A_96_p044346 | | 3.56 | 0.76 | peroxidase 12 (PER12) |
| A_96_p029251 | | 3.53 | 0.73 | Cellulose synthase-like protein G1 |
| A_96_p232449 | | 3.51 | 1.31 | ser/thr protein phosphatase , expressed |
| A_96_p188934 | | 3.48 | 0.79 | peroxidase 12 (PER12) |
| A_96_p073694 | | 3.46 | 0.81 | chloroplast atp synthase chain precursor |
| A_96_p040486 | | 3.46 | 0.77 | zinc finger (C3HC4-type RING finger) |
| A_96_p128627 | | 3.43 | 0.68 | oxidoreductase |
| A_96_p020146 | | 3.29 | 0.89 | L-asparaginase / L-asparagine amidohydrolase |
| A_96_p259463 | | 3.27 | 0.98 | Ribulose bisphosphate carboxylase small chain 3A/3C, chloroplastic |
| A_96_p024661 | | 3.25 | 0.70 | ABC transporter |
| A_96_p147991 | | 3.20 | 1.09 | ubiquitin-conjugating enzyme 4 (UBC4) |
| A_96_p219594 | | 3.20 | 1.03 | F-box , similar to SKP1 interacting partner 2 (SKIP2) |
| A_96_p126597 | | 3.20 | 1.37 | SYP41 (SYNTAXIN OF PLANTS 41) |
| A_96_p121837 | | 3.18 | 0.98 | monovalent cation:proton antiporter family 2 (CPA2) |
| A_96_p256097 | | 3.01 | 0.67 | pectate lyase |
| A_96_p127167 | | 2.99 | 1.21 | L-asparaginase / L-asparagine amidohydrolase |
| A_96_p224009 | | 2.99 | 1.52 | senescence-associated protein-related |
| A_96_p109797 | | 2.99 | 0.93 | nitrate transporter, putative |
| A_96_p033296 | | 2.97 | 0.91 | zinc finger (C3HC4-type RING finger) |
| A_96_p126767 | | 2.97 | 0.97 | NAC SECONDARY WALL THICKENING PROMOTING FACTOR1 |
| A_96_p004291 | | 2.91 | 0.73 | catalytic LigB subunit of aromatic ring-opening dioxygenase |
| A_96_p195964 | | 2.91 | 0.86 | heterotrophic ferredoxin 2 |
| A_96_p188939 | | 2.89 | 0.66 | disease resistance-responsive protein-related |
| A_96_p123392 | | 2.87 | 1.49 | copper-binding |
| A_96_p087764 | | 2.85 | 0.66 | Oxygen-evolving enhancer protein 1, chloroplast precursor (OEE1) |
| A_96_p063096 | | 2.81 | 0.61 | chloroplast-encoded gene for beta subunit of ATP synthase |
| A_96_p088954 | | 2.71 | 0.61 | photosystem ii 22 kda protein, chloroplast precursor |
| A_96_p076479 | | 2.85 | 1.18 | glutathione s-transferase |
| A_96_p028921 | | 2.83 | 1.29 | cyclin-dependent protein kinase activating kinase |
| A_96_p089844 | | 2.83 | 1.21 | polyubiquitin |
| A_96_p214954 | | 2.83 | 0.79 | F-box (FBL12) |
| A_96_p055126 | | 2.83 | 0.78 | MYB43 (myb domain protein 43) |
| A_96_p059421 | | 2.83 | 0.93 | exostosin |
| A_96_p063096 | | 2.81 | 0.61 | chloroplast-encoded gene for beta subunit of ATP synthase |
| A_96_p024081 | | 2.79 | 0.95 | similar to disease resistance protein (CC-NBS-LRR class), putative |
| A_96_p167849 | | 2.79 | 1.41 | Cytochrome P450 714A1 (CYP714A1) |
| A_96_p033266 | | 2.79 | 1.40 | dormancy-associated protein, putative (DRM1) |
| A_96_p124507 | | 2.77 | 0.96 | malonyl-CoA decarboxylase |
| A_96_p094414 | | 2.77 | 0.66 | BT1 (BTB and TAZ domain protein 1) |
| A_96_p010666 | | 2.77 | 0.93 | Transmembrane protein Tmp21 precursor |
| A_96_p047756 | | 2.75 | 0.99 | transferase |
| A_96_p013011 | | 2.75 | 0.79 | cupin |
| A_96_p033766 | | 2.73 | 1.17 | zinc finger (Ran-binding) |
| A_96_p088954 | | 2.71 | 0.61 | photosystem ii 22 kda protein, chloroplast precursor |
| A_96_p085815 | | 2.71 | 0.95 | ST (steroid sulfotransferase) |
| A_96_p128507 | | 2.71 | 0.97 | pectinesterase |
| A_96_p089514 | | 2.71 | 1.11 | bZIP transcription factor (OBF4) |
| A_96_p126972 | | 2.71 | 0.67 | indole-3-acetic acid amido synthetase |
| A_96_p163261 | | 2.69 | 0.62 | lipase class 3 |
| A_96_p064356 | | 2.69 | 0.72 | WRKY20 (WRKY transcription factor) |
| A_96_p025456 | | 2.68 | 0.65 | leucine-rich repeat |
| A_96_p055381 | | 2.68 | 1.41 | copper chaperone (CCH)-related |
| A_96_p050851 | | 2.68 | 1.57 | copper-binding |
| A_96_p060811 | | 2.66 | 1.00 | 7-dehydrocholesterol reductase |
| A_96_p114742 | | 2.66 | 0.89 | trypsin and protease inhibitor |
| A_96_p167854 | | 2.66 | 0.66 | dihydroflavonol 4-reductase |
| A_96_p022336 | | 2.66 | 0.84 | GAUT15 (Galacturonosyltransferase 15) |
| A_96_p049886 | | 2.64 | 0.76 | exostosin |
| A_96_p223189 | | 2.64 | 0.96 | ABC transporter |
| A_96_p038036 | | 2.62 | 0.72 | glycine-rich protein |
| A_96_p018356 | | 2.62 | 1.01 | protease-associated (PA) domain-containing protein |
| A_96_p009176 | | 2.62 | 1.79 | auxin and ethylene responsive GH3-like protein |
| A_96_p011546 | | 2.62 | 1.27 | ATPase 1, plasma membrane-type, putative |
| A_96_p247187 | | 2.57 | 1.69 | H+-transporting two-sector ATPase, putative |
| A_96_p006746 | | 2.57 | 1.00 | leucine-rich repeat protein |
| A_96_p127682 | | 2.55 | 1.45 | nodulin MtN3 |
| A_96_p150101 | | 2.52 | 0.96 | polygalacturonase, putative |
| A_96_p214419 | | 2.52 | 0.89 | Fasciclin-like arabinogalactan protein (FLA1) |
| A_96_p173659 | | 2.51 | 0.62 | 2-oxoglutarate-dependent dioxygenase, putative |
| A_96_p176549 | | 2.50 | 1.98 | phenylalanine ammonia-lyase (PAL1) |
| A_96_p173809 | | 2.47 | 1.12 | glycoside hydrolase 19 |
| A_96_p145426 | | 2.47 | 1.33 | ABC transporter |
| A_96_p152301 | | 2.46 | 1.55 | short-chain dehydrogenase/reductase (SDR) |
| A_96_p057001 | | 2.45 | 1.12 | GCN5-related N-acetyltransferase (GNAT) |
| A_96_p070769 | | 2.44 | 1.54 | proton-dependent oligopeptide transport (POT) |
| A_96_p224080 | | 2.42 | 1.00 | transparent testa glabra 1 protein (TTG1) |
| A_96_p099334 | | 2.41 | 1.79 | heat shock protein class i |
| A_96_p183169 | | 2.40 | 0.91 | ABC transporter |
| A_96_p018746 | | 2.37 | 1.96 | kelch repeat-containing protein |
| A_96_p018746 | | 2.37 | 1.59 | kelch repeat-containing protein |
| A_96_p059266 | | 2.37 | 1.24 | caleosin-related |
| A_96_p229784 | | 2.37 | 1.56 | isoflavone reductase |
| A_96_p039401 | | 2.36 | 0.99 | calcium antiporter CAX1 |
| A_96_p052336 | | 2.35 | 1.72 | leucoanthocyanidin dioxygenase-like protein |
| A_96_p093779 | | 2.35 | 0.75 | ALPHA-DOX1 (ALPHA-DIOXYGENASE 1) |
| A_96_p003731 | | 2.34 | 1.30 | XF1 (SQUALENE EPOXIDASE 1) |
| A_96_p077729 | | 2.34 | 1.98 | HEAT SHOCK PROTEIN 18.2 |
| A_96_p169899 | | 2.32 | 1.64 | ethylene-responsive |
| A_96_p074814 | | 2.32 | 0.79 | PAP27 (purple acid phosphatase 27) |
| A_96_p055041 | | 2.31 | 1.42 | chalcone-flavanone isomerase |
| A_96_p098659 | | 2.31 | 1.47 | cytochrome b5 domain-containing protein |
| A_96_p224669 | | 2.31 | 1.94 | ABC transporter |
| A_96_p045071 | | 2.29 | 1.90 | DnaJ-like protein |
| A_96_p040441 | | 2.28 | 0.88 | senescence-associated protein-related |
| A_96_p022176 | | 2.27 | 0.73 | 2-oxoglutarate-dependent dioxygenase, putative |
| A_96_p091469 | | 2.27 | 0.87 | DWF1 (DIMINUTO 1) |
| A_96_p144576 | | 2.27 | 1.99 | phenylalanine ammonia-lyase, putative (PAL4) |
| A_96_p071614 | | 2.27 | 0.91 | auxin-responsive protein, putative |
| A_96_p070669 | | 2.27 | 1.90 | peptidyl-prolyl cis-trans isomerase cyclophilin-type |
| A_96_p197194 | | 2.26 | 1.53 | arachidonic acid-induced dea1 |
| A_96_p003816 | | 2.25 | 1.21 | ATB5-A (Cytochrome b5 A) |
| A_96_p219549 | | 2.24 | 1.84 | DNAJ heat shock protein, putative (J3) |
| A_96_p159816 | | 2.23 | 0.69 | STE1 (STEROL 1); C-5 sterol desaturase |
| A_96_p225824 | | 2.22 | 1.09 | embryogenesis-associated protein-related |
| A_96_p027451 | | 2.21 | 1.08 | chalcone and stilbene synthase |
| A_96_p000761 | | 2.21 | 1.29 | nodulin MtN21 |
| A_96_p128532 | | 2.21 | 1.76 | caffeoyl-CoA 3-O-methyltransferase, putative |
| A_96_p199339 | | 2.20 | 1.32 | GLIP5 (GDSL-motif lipase 5) |
| A_96_p067396 | | 2.19 | 1.39 | ZIP1 (ZINC TRANSPORTER 1 PRECURSOR) |
| A_96_p202479 | | 2.18 | 1.78 | auxin efflux carrier |
| A_96_p146326 | 2.18 | 1.78 | chitinase |  |
| A_96_p017311 | 2.18 | 1.34 | DNAJ heat shock N-terminal domain-containing protein |  |
| A_96_p258377 | | 2.18 | 1.87 | amino acid transporter |
| A_96_p112307 | | 2.17 | 1.99 | plastocyanin-like domain-containing protein |
| A_96_p246662 | | 2.16 | 1.62 | ABC transporter |
| A_96_p028976 | | 2.15 | 1.53 | MLO1 (MILDEW RESISTANCE LOCUS O 1) |
| A_96_p093529 | | 2.14 | 0.90 | XYL4 (beta-xylosidase 4) |
| A_96_p048621 | | 2.14 | 0.88 | peroxidase |
| A_96_p101869 | | 2.12 | 1.43 | WD-40 repeat |
| A_96_p229754 | | 2.11 | 0.67 | pentatricopeptide (PPR) repeat-containing protein (PPR) |
| A_96_p097214 | | 2.11 | 1.00 | DNAJ heat shock |
| A_96_p123682 | | 2.10 | 1.64 | cellulose synthase-like E1 |
| A_96_p114142 | | 2.09 | 0.88 | heat shock |
| A_96_p028831 | | 2.08 | 1.79 | PAP26 (purple acid phosphatase 26) |
| A_96_p018806 | | 2.07 | 1.02 | cyclin-dependent protein kinase |
| A_96_p075024 | | 2.07 | 0.97 | allergen V5/Tpx-1-related |
| A_96_p062631 | | 2.07 | 1.44 | amino acid transporter |
| A_96_p121202 | | 2.06 | 1.23 | disease resistance protein (NBS-LRR class), putative |
| A_96_p048736 | | 2.05 | 0.87 | EFE (ETHYLENE FORMING ENZYME) |
| A_96_p184844 | | 2.05 | 1.64 | oxidoreductase |
| A_96_p015021 | | 2.05 | 0.91 | NAC1 |
| A_96_p027391 | | 2.05 | 0.97 | 15.7 kDa class I-related small heat shock protein-like (HSP15.7-CI) |
| A_96_p055066 | | 2.04 | 1.34 | ELI3-1 (ELICITOR-ACTIVATED GENE 3) |
| A_96_p017146 | | 2.04 | 1.05 | NIC2 (NICOTINAMIDASE 2) |
| A_96_p098454 | | 2.03 | 1.11 | nodulin MtN21 |
| A_96_p210029 | | 2.02 | 1.22 | HEAT SHOCK PROTEIN 101 |
| A_96_p144801 | | 2.01 | 1.98 | high-affinity nitrate transporter |
| A_96_p018351 | | 2.01 | 1.44 | VHA-A3 (VACUOLAR PROTON ATPASE A3) |
| A_96_p054436 | | 2.01 | 1.44 | trypsin and protease inhibitor |
| A_96_p211794 | | 2.01 | 1.88 | KUP7 (K+ uptake permease 7) |
| A_96_p013363 | | 2.01 | 1.09 | hero resistance protein 3 homologue |
| A_96_p156296 | | 2.00 | 1.46 | calcium:sodium antiporter (CAX7) |
| Leaves of tomato plants treated with either EM 0.1 mL L^-1^ or EM 1 mL L^-1^: ***up-regulation in EM1 and down-regulation or no effect in EM0.1*** | | | | |
| **Agilent ID** | | **Fold change** | | **Annotation** |
|  | | *EM 0.1 mL L^-1^* | *EM 1 mL L^-1^* |  |
| A_96_p203664 | | 0.98 | 321.80 | Pathogenesis-related protein Bet v |
| A_96_p056646 | | 1.31 | 69.07 | expansin like B1 |
| A_96_p223439 | | 1.54 | 63.51 | In2-1 protein, putative |
| A_96_p015511 | | 0.24 | 56.78 | oxidoreductase |
| A_96_p124062 | | 0.68 | 21.80 | late embryogenesis abundant domain-containing protein |
| A_96_p059631 | | 1.76 | 19.20 | syntaxin-related protein (SYR1) |
| A_96_p108232 | | 1.88 | 17.89 | protein kinase |
| A_96_p232679 | | 1.46 | 16.83 | CAM1 (CALMODULIN 1) |
| A_96_p031476 | | 1.32 | 15.26 | DC1 domain-containing protein |
| A_96_p208614 | | 1.11 | 15.07 | 2-oxoglutarate-dependent dioxygenase, putative |
| A_96_p138737 | | 1.07 | 14.53 | leucine-rich repeat protein |
| A_96_p108512 | | 1.08 | 14.22 | BETA-TIP (BETA-TONOPLAST INTRINSIC PROTEIN) |
| A_96_p045391 | | 1.35 | 12.24 | lob domain protein 1, putative |
| A_96_p043716 | | 0.56 | 11.38 | coatomer protein complex |
| A_96_p124427 | | 0.97 | 11.27 | Glutathione S-transferase (class tau) |
| A_96_p008266 | | 1.49 | 10.99 | UDP-glucoronosyl/UDP-glucosyl transferase |
| A_96_p187314 | | 1.26 | 9.18 | Chaperone protein dnaJ (40 kDa heat shock chaperone protein) |
| A_96_p042851 | | 1.20 | 7.92 | calcium-binding EF hand |
| A_96_p199789 | | 1.38 | 6.99 | putative thaumatin-like protein |
| A_96_p057541 | | 1.65 | 6.48 | aspartyl protease |
| A_96_p072125 | | 0.89 | 6.35 | Glutathione S-transferase (class tau) |
| A_96_p127932 | | 0.94 | 6.27 | myb transcription factor (MYB15) |
| A_96_p160186 | | 0.17 | 6.12 | transferase |
| A_96_p143696 | | 1.76 | 6.11 | GAMMA-VPE (Vacuolar processing enzyme gamma) |
| A_96_p155321 | | 1.91 | 6.11 | cysteine-type endopeptidase |
| A_96_p229929 | | 1.84 | 6.02 | 2-oxoglutarate-dependent dioxygenase, putative |
| A_96_p041346 | | 1.77 | 5.80 | UDP-glycosyltransferase |
| A_96_p081064 | | 1.16 | 5.79 | UDP-glycosyltransferase |
| A_96_p044431 | | 1.19 | 5.65 | GTe, putative |
| A_96_p139902 | | 1.05 | 5.64 | S-locus lectin protein kinase |
| A_96_p043681 | | 1.02 | 5.62 | AMP-dependent synthetase and ligase |
| A_96_p144076 | | 1.90 | 5.43 | AAA-type ATPase |
| A_96_p136142 | | 0.96 | 5.39 | 2-oxoglutarate-dependent dioxygenase, putative |
| A_96_p139507 | | 1.00 | 5.24 | leucine-rich repeat |
| A_96_p155256 | | 1.22 | 5.23 | 1-aminocyclopropane-1-carboxylate oxidase 4 |
| A_96_p078694 | | 1.64 | 5.22 | 17.8 kDa class I heat shock protein (HSP17.8-CI) |
| A_96_p194744 | | 1.09 | 5.18 | leucine-rich repeat |
| A_96_p043761 | | 0.86 | 5.11 | harpin-induced protein-related |
| A_96_p124592 | | 1.21 | 5.07 | ammonium transmembrane transporter |
| A_96_p044396 | | 1.67 | 5.03 | AAA-type ATPase |
| A_96_p000136 | | 1.88 | 5.00 | chitinase, class ii precursor |
| A_96_p239438 | | 1.03 | 4.95 | serine carboxypeptidase |
| A_96_p145716 | | 0.89 | 4.87 | aspartyl protease |
| A_96_p056861 | | 1.94 | 4.86 | ammonium transmembrane transporter (AMT1.1) |
| A_96_p049291 | | 1.61 | 4.80 | DNAJ heat shock N-terminal domain-containing protein |
| A_96_p033501 | | 1.00 | 4.78 | glutathione-conjugate transporter (MRP4) |
| A_96_p043421 | | 0.93 | 4.75 | putative receptor-like serine-threonine protein kinase |
| A_96_p032731 | | 1.08 | 4.75 | ankyrin repeat |
| A_96_p120347 | | 1.17 | 4.68 | carboxylesterase |
| A_96_p143756 | | 1.28 | 4.56 | lectin protein kinase |
| A_96_p035821 | | 0.95 | 4.47 | leucine-rich repeat |
| A_96_p056726 | | 1.43 | 4.40 | ribulose bisphosphate carboxylase small chain 3a/3c, chloroplast |
| A_96_p093764 | | 1.57 | 4.39 | ERF/AP2 transcription factor. |
| A_96_p125732 | | 1.71 | 4.34 | leucine-rich repeat |
| A_96_p237232 | | 1.06 | 4.33 | CPL17 (serine carboxypeptidase-like 17) |
| A_96_p017006 | | 0.99 | 4.31 | disease resistance |
| A_96_p196839 | | 1.23 | 4.31 | omega-6 fatty acid desaturase |
| A_96_p134862 | | 1.78 | 4.30 | wound-responsive protein-related |
| A_96_p215764 | | 1.65 | 4.28 | Late embryogenesis abundant protein |
| A_96_p092854 | | 1.92 | 4.26 | TAP-like ABC transporter |
| A_96_p117947 | | 1.37 | 4.25 | XLG1 (EXTRA-LARGE G-PROTEIN 1) |
| A_96_p072339 | | 1.05 | 4.20 | cyclic nucleotide-gated channel (CNGC1) |
| A_96_p050681 | | 1.00 | 4.16 | ubiquitin interaction motif-containing protein |
| A_96_p248612 | | 1.13 | 4.16 | lectin protein kinase |
| A_96_p137202 | | 1.54 | 4.13 | phenazine biosynthesis |
| A_96_p191269 | | 1.68 | 4.12 | cysteine proteinase, putative |
| A_96_p122927 | | 1.38 | 4.11 | amino acid transporter |
| A_96_p223239 | | 1.04 | 4.08 | heat shock protein binding |
| A_96_p140177 | | 1.55 | 4.06 | photoassimilate-responsive protein-related |
| A_96_p112162 | | 1.93 | 4.06 | protein kinase |
| A_96_p012186 | | 0.91 | 4.04 | beta-carotene hydroxylase |
| A_96_p127992 | | 1.76 | 4.04 | leucine-rich repeat transmembrane protein kinase, putative |
| A_96_p039471 | | 0.94 | 4.04 | 18.1 kDa class I heat shock protein (HSP18.1-CI) |
| A_96_p241334 | | 1.88 | 4.02 | AATP1 (AAA-ATPASE 1) |
| A_96_p003951 | | 1.03 | 4.00 | S2P-like putative metalloprotease |
| A_96_p078504 | | 1.32 | 2.58 | myb 44 transcription factor |
| A_96_p097479 | | 1.09 | 2.57 | isoflavone reductase, putative |
| A_96_p048361 | | 0.54 | 2.57 | mitotic checkpoint |
| A_96_p109937 | | 1.17 | 2.55 | DNAJ heat shock N-terminal domain-containing protein |
| A_96_p064186 | | 0.97 | 2.55 | basic helix-loop-helix (bHLH) |
| A_96_p062346 | | 1.34 | 2.55 | Pathogenesis-related protein Bet v I |
| A_96_p061251 | | 0.83 | 2.55 | peptide chain release factor, putative |
| A_96_p115297 | | 0.96 | 2.53 | RING-H2 zinc finger protein |
| A_96_p166734 | | 0.86 | 2.53 | alpha-amylase |
| A_96_p125202 | | 0.98 | 2.53 | beta expansin precursor |
| A_96_p200309 | | 1.26 | 2.53 | AMP-dependent synthetase and ligase |
| A_96_p211499 | | 0.95 | 2.51 | Myb-like DNA-binding domain-containing protein |
| A_96_p018116 | | 1.34 | 2.51 | beta-fructofuranosidase, putative |
| A_96_p145341 | | 1.00 | 2.51 | EXGT-A3 (endo-xyloglucan transferase A3 |
| A_96_p066901 | | 0.61 | 2.51 | amidase |
| A_96_p177220 | | 1.13 | 2.51 | EIF2 GAMMA (eukaryotic translation initiation factor 2 gamma subunit) |
| A_96_p163581 | | 0.47 | 2.51 | Kinase |
| A_96_p221584 | | 0.59 | 2.51 | myosin heavy chain MYA2 |
| A_96_p159666 | | 0.76 | 2.51 | mitotic spindle checkpoint protein, putative (MAD2) |
| A_96_p108522 | | 0.78 | 2.50 | IAA-amido synthases |
| A_96_p065131 | | 0.55 | 2.50 | nitroreductase |
| A_96_p247297 | | 0.94 | 2.50 | ATPAP15/PAP15 (purple acid phosphatase 15) |
| A_96_p202064 | | 0.37 | 2.50 | phragmoplastin, putative |
| A_96_p161356 | | 0.76 | 2.50 | C/VIF2 (CELL WALL / VACUOLAR INHIBITOR OF FRUCTOSIDASE 2) |
| A_96_p136137 | | 1.02 | 2.50 | ABC transporter |
| A_96_p003796 | | 1.78 | 2.48 | armadillo/beta-catenin repeat |
| A_96_p037576 | | 1.27 | 2.48 | 2,3-biphosphoglycerate-independent phosphoglycerate mutase |
| A_96_p112197 | | 0.63 | 2.48 | glycosyl hydrolase family 3 protein |
| A_96_p207859 | | 1.32 | 2.46 | beta-fructofuranosidase, putative |
| A_96_p031811 | | 0.97 | 2.46 | glycosyltransferase |
| A_96_p226684 | | 0.94 | 2.46 | oxidoreductase, 2OG-Fe(II) oxygenase |
| A_96_p115942 | | 0.45 | 2.46 | glutamine amidotransferase |
| A_96_p230429 | | 1.46 | 2.46 | curculin-like (mannose-binding) lectin |
| A_96_p203209 | | 0.61 | 2.46 | similar to kinesin motor protein (kin2) |
| A_96_p133197 | | 0.58 | 2.45 | peroxidase 40 (PER40) (P40) |
| A_96_p073989 | | 0.48 | 2.45 | SPA2 (SPA1-RELATED 2) |
| A_96_p095904 | | 1.35 | 2.45 | ZFN1 (ZINC FINGER PROTEIN 1) |
| A_96_p121912 | | 1.93 | 2.45 | histone-like transcription factor (CBF/NF-Y) |
| A_96_p066301 | | 1.14 | 2.45 | T521-B-like |
| A_96_p125327 | | 0.96 | 2.45 | plus-end-directed microtubule motor |
| A_96_p205154 | | 0.94 | 2.45 | MATE efflux |
| A_96_p109182 | | 0.55 | 2.43 | dehydration-responsive |
| A_96_p224474 | | 1.07 | 2.43 | PHD finger |
| A_96_p152536 | | 0.96 | 2.43 | basic helix-loop-helix (bHLH) |
| A_96_p229044 | | 0.98 | 2.43 | curculin-like (mannose-binding) lectin |
| A_96_p191799 | | 0.94 | 2.43 | PIP (proline iminopeptidase) |
| A_96_p059766 | | 0.87 | 2.43 | F-box |
| A_96_p084119 | | 0.78 | 2.43 | cyclin-dependent protein kinase regulator |
| A_96_p068786 | | 0.99 | 2.43 | nitrate transporter (NTP2) |
| A_96_p066201 | | 1.04 | 2.41 | lrr receptor-like kinase |
| A_96_p074664 | | 1.04 | 2.41 | leucine-rich repeat transmembrane protein kinase, putative |
| A_96_p112857 | | 0.96 | 2.41 | leucine-rich repeat |
| A_96_p220784 | | 1.12 | 2.41 | phytochelatin synthetase |
| A_96_p169914 | | 1.24 | 2.41 | zinc finger (B-box type) |
| A_96_p131797 | | 0.89 | 2.41 | zinc finger (CCCH-type) |
| A_96_p223109 | | 0.97 | 2.41 | ER lumen protein retaining receptor |
| A_96_p136892 | | 1.44 | 2.41 | ERF1-3 (EUKARYOTIC RELEASE FACTOR 1-3) |
| A_96_p019251 | | 0.97 | 2.41 | ER lumen protein retaining receptor |
| A_96_p227054 | | 1.67 | 2.41 | cysteine-type peptidase |
| A_96_p119457 | | 0.95 | 2.39 | AMP-dependent synthetase and ligase |
| A_96_p069369 | | 0.76 | 2.39 | URE (UREASE); |
| A_96_p248207 | | 0.90 | 2.39 | oxidoreductase, 2OG-Fe(II) oxygenase |
| A_96_p138552 | | 0.92 | 2.39 | ERF3 (ETHYLENE RESPONSIVE ELEMENT BINDING FACTOR 3) |
| A_96_p248082 | | 1.33 | 2.39 | ethylene-responsive transcription factor 4 |
| A_96_p246280 | | 1.56 | 2.39 | thioredoxin h |
| A_96_p199964 | | 1.87 | 2.39 | SNF2 domain-containing protein |
| A_96_p134088 | | 0.98 | 2.39 | ERF1-3 (EUKARYOTIC RELEASE FACTOR 1-3) |
| A_96_p126672 | | 1.06 | 2.39 | kinesin motor protein-related |
| A_96_p122730 | | 1.08 | 2.39 | lim domain protein plim-2 |
| A_96_p124222 | | 1.06 | 2.38 | F-box |
| A_96_p038026 | | 0.89 | 2.38 | chloroplast small heat shock protein |
| A_96_p123302 | | 1.21 | 2.38 | zinc finger (ZPR1-type) |
| A_96_p231384 | | 1.85 | 2.38 | carbohydrate binding / kinase |
| A_96_p048451 | | 1.10 | 2.36 | auxin-responsive |
| A_96_p060446 | | 1.46 | 2.36 | CER1 protein, putative (WAX2) |
| A_96_p113922 | | 0.98 | 2.36 | THE1 (THESEUS1) |
| A_96_p004576 | | 0.92 | 2.36 | fimbrin-like protein, putative |
| A_96_p059081 | | 1.08 | 2.36 | ankyrin repeat |
| A_96_p172759 | | 1.20 | 2.36 | CIP111 (CAM INTERACTING PROTEIN 111) |
| A_96_p186719 | | 1.07 | 2.35 | soluble starch synthase 3 |
| A_96_p131252 | | 0.87 | 2.35 | PCK2/PEPCK (PHOSPHOENOLPYRUVATE CARBOXYKINASE 2) |
| A_96_p122747 | | 0.65 | 2.35 | 3-dehydroquinate dehydratase |
| A_96_p103069 | | 0.78 | 2.35 | pollen Ole e 1 allergen and extensin |
| A_96_p103619 | | 0.88 | 2.35 | ribose-phosphate pyrophosphokinase 2 |
| A_96_p214249 | | 0.79 | 2.35 | AIL6 (AINTEGUMENTA-LIKE 6) |
| A_96_p196399 | | 0.90 | 2.35 | glycerol-3-phosphate transporter, putative |
| A_96_p183439 | | 1.23 | 2.33 | peroxidase, putative |
| A_96_p187144 | | 1.74 | 2.33 | expansin precursor |
| A_96_p143996 | | 1.03 | 2.33 | clathrin adaptor complexes medium subunit |
| A_96_p065216 | | 1.14 | 2.33 | ubiquitin-related |
| A_96_p018136 | | 0.95 | 2.33 | proton-dependent oligopeptide transport (POT) |
| A_96_p037491 | | 1.28 | 2.31 | DNAJ heat shock N-terminal domain-containing protein (J11) |
| A_96_p077739 | | 1.65 | 2.31 | universal stress protein (USP) |
| A_96_p078489 | | 1.25 | 2.31 | GAUT1/LGT1 (Galacturonosyltransferase 1) |
| A_96_p039416 | | 1.10 | 2.31 | ShTK domain-containing protein |
| A_96_p069619 | | 0.77 | 2.31 | CIA2 \| CIA2 (CHLOROPLAST IMPORT APPARATUS 2) |
| A_96_p199619 | | 0.67 | 2.31 | ATP-dependent helicase |
| A_96_p023286 | | 0.94 | 2.31 | SCPL25 (serine carboxypeptidase-like 25) |
| A_96_p107504 | | 1.09 | 2.31 | GI (GIGANTEA) |
| A_96_p070804 | | 1.12 | 2.31 | no apical meristem (NAM) |
| A_96_p144081 | | 1.43 | 2.30 | CKX7 (CYTOKININ OXIDASE 7) |
| A_96_p201369 | | 1.00 | 2.30 | SOUL heme-binding |
| A_96_p088639 | | 1.33 | 2.30 | adenine phosphoribosyltransferase-like |
| A_96_p246912 | | 1.06 | 2.30 | RNA polymerase Rpb3 |
| A_96_p024001 | | 1.78 | 2.30 | ILR3 (IAA-LEUCINE RESISTANT3) |
| A_96_p043276 | | 0.87 | 2.30 | P-P-bond-hydrolysis-driven protein transmembrane transporter |
| A_96_p206884 | | 1.14 | 2.30 | serine-type peptidase/ trypsin |
| A_96_p237677 | | 1.53 | 2.28 | UBC9 (UBIQUITIN CONJUGATING ENZYME 9) |
| A_96_p139717 | | 0.87 | 2.28 | DNAJ heat shock protein, putative (J3) |
| A_96_p227899 | | 1.35 | 2.28 | protein kinase |
| A_96_p077474 | | 0.95 | 2.28 | NDR1 (NON RACE-SPECIFIC DISEASE RESISTANCE 1) |
| A_96_p106404 | | 1.56 | 2.28 | pollen Ole e 1 allergen and extensin |
| A_96_p182229 | | 1.78 | 2.28 | Cytochrome P450 76G1 (CYP76G1) |
| A_96_p124317 | | 1.70 | 2.28 | zinc finger (GATA type) |
| A_96_p203769 | | 1.57 | 2.28 | transcriptional regulator-related |
| A_96_p045636 | | 1.38 | 2.28 | transcriptional co-activator-related |
| A_96_p124502 | | 1.06 | 2.28 | kinesin motor protein-related |
| A_96_p231269 | | 0.77 | 2.28 | proton-dependent oligopeptide transport (POT) |
| A_96_p178139 | | 1.76 | 2.27 | DNAJ heat shock N-terminal domain-containing protein |
| A_96_p186124 | | 1.44 | 2.27 | centromeric histone H3 HTR12 (HTR12) |
| A_96_p208219 | | 1.18 | 2.27 | gamma-glutamyl hydrolase |
| A_96_p224059 | | 1.54 | 2.27 | penicillin-binding protein |
| A_96_p028581 | | 0.87 | 2.27 | ubiquitin thiolesterase |
| A_96_p191039 | | 1.61 | 2.27 | chromosome-associated kinesin, putative |
| A_96_p154411 | | 1.45 | 2.27 | nodulin |
| A_96_p203364 | | 1.00 | 2.27 | potassium channel tetramerisation domain-containing protein |
| A_96_p036756 | | 1.20 | 2.25 | HSP91 (Heat shock protein 91) |
| A_96_p124607 | | 1.38 | 2.25 | calcium-transporting ATPase |
| A_96_p110812 | | 0.69 | 2.25 | FRS2 (FAR1-RELATED SEQUENCE 2) |
| A_96_p139792 | | 1.54 | 2.25 | xyloglucan:xyloglucosyl transferase, putative |
| A_96_p187534 | | 1.62 | 2.25 | zinc finger (MYND type) |
| A_96_p208659 | | 1.34 | 2.25 | RNA recognition motif (RRM)-containing protein |
| A_96_p134412 | | 0.94 | 2.25 | tRNA synthetase class I (C) |
| A_96_p136702 | | 1.49 | 2.25 | OXA1 (Oxidase assembly 1) |
| A_96_p062286 | | 1.78 | 2.25 | XCP1 (XYLEM CYSTEINE PEPTIDASE 1) |
| A_96_p019091 | | 1.54 | 2.25 | purine permease-related |
| A_96_p247362 | | 0.87 | 2.25 | GAMMA-TIP (Tonoplast intrinsic protein (TIP) gamma) |
| A_96_p068016 | | 1.60 | 2.23 | phytochrome kinase substrate-related |
| A_96_p148481 | | 1.55 | 2.23 | lipid transfer protein-related |
| A_96_p060451 | | 1.23 | 2.23 | Cytochrome P450 71B36 (CYP71B36) |
| A_96_p224919 | | 1.67 | 2.23 | zinc finger (B-box type) |
| A_96_p050276 | | 1.49 | 2.23 | zinc finger (FYVE type) |
| A_96_p017456 | | 1.57 | 2.23 | translocase inner membrane subunit 23-2 |
| A_96_p212554 | | 1.78 | 2.23 | RLK (RECEPTOR LECTIN KINASE) |
| A_96_p097769 | | 1.05 | 2.23 | Ku80 |
| A_96_p182814 | | 1.00 | 2.22 | WRKY72 (WRKY DNA-binding protein 72) |
| A_96_p119827 | | 0.87 | 2.22 | UDP-glucose 6-dehydrogenase, putative |
| A_96_p107864 | | 0.99 | 2.22 | ASP1 (ASPARTATE AMINOTRANSFERASE 1) |
| A_96_p139612 | | 1.25 | 2.22 | EMB1967 (EMBRYO DEFECTIVE 1967) |
| A_96_p108737 | | 1.36 | 2.22 | RNA recognition motif (RRM)-containing protein |
| A_96_p092974 | | 0.79 | 2.22 | ribosomal protein S8e |
| A_96_p231654 | | 0.65 | 2.22 | cell division cycle |
| A_96_p251132 | | 1.33 | 2.22 | protein kinase |
| A_96_p235775 | | 0.77 | 2.20 | zinc finger (C3HC4-type RING finger) |
| A_96_p170494 | | 0.70 | 2.20 | GBF1 (G-box binding factor 1) |
| A_96_p158431 | | 1.78 | 2.20 | glutamate receptor (GLR3.3) |
| A_96_p023436 | | 1.48 | 2.20 | eucine-rich repeat transmembrane protein kinase, putative |
| A_96_p073594 | | 1.50 | 2.20 | beta-fructofuranosidase, putative |
| A_96_p044356 | | 1.55 | 2.20 | thioredoxin |
| A_96_p173974 | | 0.93 | 2.20 | DNA gyrase subunit A |
| A_96_p038096 | | 1.81 | 2.20 | CHR5 (chromatin remodeling 5) |
| A_96_p220849 | | 1.76 | 2.20 | inositol-polyphosphate 5-phosphatase |
| A_96_p021976 | | 1.07 | 2.20 | kinesin motor protein-related |
| A_96_p109647 | | 1.77 | 2.20 | kinesin motor protein-related |
| A_96_p219179 | | 0.96 | 2.19 | zinc finger (C3HC4-type RING finger) |
| A_96_p169619 | | 1.58 | 2.19 | F-box (FBL17) |
| A_96_p203044 | | 1.14 | 2.19 | phototropic-responsive NPH3 |
| A_96_p198569 | | 0.69 | 2.19 | oxidoreductase, 2OG-Fe(II) oxygenase |
| A_96_p084989 | | 0.95 | 2.19 | ribosomal protein L22 |
| A_96_p181604 | | 0.78 | 2.19 | ceramide kinase-related |
| A_96_p074674 | | 0.65 | 2.19 | AATP1 (AAA-ATPASE 1) |
| A_96_p158696 | | 1.43 | 2.19 | zinc finger (C3HC4-type RING finger) |
| A_96_p207239 | | 0.87 | 2.17 | transcription factor jumonji (jmjC) domain-containing protein |
| A_96_p062461 | | 1.65 | 2.17 | AP2 domain-containing transcription factor, putative |
| A_96_p005081 | | 1.66 | 2.17 | ATPase epsilon subunit |
| A_96_p144591 | | 1.23 | 2.17 | MIOX4 (MYO-INOSITOL OXYGENASE 4) |
| A_96_p103064 | | 1.45 | 2.17 | diacylglycerol kinase |
| A_96_p188449 | | 1.88 | 2.17 | acidic endochitinase (CHIB1) |
| A_96_p200849 | | 1.67 | 2.17 | disease resistance protein (CC-NBS-LRR class), putative |
| A_96_p023641 | | 1.87 | 2.17 | pollen Ole e 1 allergen and extensin |
| A_96_p205814 | | 1.58 | 2.17 | glycosyl transferase 1 protein |
| A_96_p108517 | | 1.04 | 2.17 | alpha-N-acetylglucosaminidase |
| A_96_p200894 | | 1.23 | 2.17 | EMB1967 \| EMB1967 (EMBRYO DEFECTIVE 1967) |
| A_96_p134377 | | 0.75 | 2.17 | PHD finger |
| A_96_p212319 | | 1.13 | 2.17 | P-P-bond-hydrolysis-driven protein transmembrane transporter |
| A_96_p094579 | | 1.18 | 2.17 | GI (GIGANTEA) |
| A_96_p161816 | | 1.09 | 2.17 | pleiotropic drug resistance protein 3 |
| A_96_p081859 | | 1.78 | 2.16 | HSP70 (heat shock protein 70) |
| A_96_p168154 | | 1.32 | 2.16 | sucrose-phosphate synthase isoform c |
| A_96_p024606 | | 1.15 | 2.16 | plastid alpha-amylase |
| A_96_p184694 | | 1.37 | 2.16 | plastocyanin-like domain-containing protein |
| A_96_p033881 | | 0.87 | 2.16 | high mobility group (HMG1/2) |
| A_96_p054876 | | 1.67 | 2.16 | armadillo/beta-catenin repeat |
| A_96_p124852 | | 1.48 | 2.16 | phosphatidylinositol-4-phosphate 5-kinase |
| A_96_p186204 | | 0.78 | 2.16 | RAN-1 (RAS RELATED NUCLEAR PROTEIN) |
| A_96_p188144 | | 0.89 | 2.16 | transducin |
| A_96_p117922 | | 1.08 | 2.16 | mitochondrial substrate carrier |
| A_96_p176284 | | 1.21 | 2.16 | rnase l inhibitor-like protein |
| A_96_p099279 | | 0.77 | 2.14 | ethylene-responsive transcriptional coactivator, putative |
| A_96_p170159 | | 0.89 | 2.14 | receptor kinase (CLV1) |
| A_96_p064986 | | 1.34 | 2.14 | calmodulin-binding protein |
| A_96_p183379 | | 1.74 | 2.14 | inositol or phosphatidylinositol kinase |
| A_96_p158611 | | 1.18 | 2.14 | basic helix-loop-helix (bHLH) |
| A_96_p191189 | | 1.06 | 2.14 | LBD4 (LOB DOMAIN-CONTAINING PROTEIN 4) |
| A_96_p128502 | | 1.48 | 2.14 | ribosomal protein S5 |
| A_96_p129832 | | 1.09 | 2.14 | protein kinase |
| A_96_p220924 | | 0.68 | 2.14 | AAA-type ATPase |
| A_96_p089844 | | 0.82 | 2.14 | UBQ3 (POLYUBIQUITIN 3) |
| A_96_p190144 | | 1.27 | 2.14 | zinc finger (RING-H2 type) protein-related |
| A_96_p002326 | | 0.77 | 2.14 | ubiquitin-specific protease, putative |
| A_96_p018796 | | 0.84 | 2.14 | GTP-binding |
| A_96_p125002 | | 1.05 | 2.14 | APC11 (anaphase-promoting complex/cyclosome 11) |
| A_96_p007526 | | 1.38 | 2.14 | cyclin-dependent protein kinase regulator |
| A_96_p059036 | | 1.44 | 2.14 | C4-dicarboxylate transporter/malic acid transport |
| A_96_p035846 | | 0.93 | 2.14 | NGC1 (CYCLIC NUCLEOTIDE GATED CHANNEL 1) |
| A_96_p027156 | | 1.58 | 2.13 | peroxidase, putative |
| A_96_p124207 | | 1.76 | 2.13 | IMK2 (INFLORESCENCE MERISTEM RECEPTOR-LIKE KINASE 2) |
| A_96_p108752 | | 1.45 | 2.13 | NTRA (NADPH-dependent thioredoxin reductase 2) |
| A_96_p018941 | | 1.09 | 2.13 | transcriptional factor B3 |
| A_96_p174664 | | 1.05 | 2.13 | SNF1-RELATED PROTEIN KINASE 2.5 |
| A_96_p191194 | | 1.10 | 2.13 | ROC2 (rotamase CyP 2) |
| A_96_p113297 | | 1.70 | 2.13 | SYP111 (syntaxin 111) |
| A_96_p051031 | | 1.56 | 2.13 | proton-dependent oligopeptide transport (POT) |
| A_96_p202834 | | 1.72 | 2.13 | ATPDR9/PDR9 (PLEIOTROPIC DRUG RESISTANCE 9) |
| A_96_p141117 | | 1.87 | 2.13 | chloride channel-like (CLC) protein, putative |
| A_96_p068237 | | 0.90 | 2.11 | leucine-rich repeat |
| A_96_p217564 | | 0.76 | 2.11 | calmodulin binding |
| A_96_p148066 | | 1.08 | 2.11 | LHCA5 (Photosystem I light harvesting complex gene 5) |
| A_96_p246947 | | 0.95 | 2.11 | SOS5 (SALT OVERLY SENSITIVE 5) |
| A_96_p094614 | | 1.88 | 2.11 | ASP2 (ASPARTATE AMINOTRANSFERASE 2) |
| A_96_p192314 | | 1.63 | 2.11 | UDP-glucosyltransferase (UGT72B1) |
| A_96_p247072 | | 1.07 | 2.11 | ARF4 (AUXIN RESPONSE FACTOR 4) |
| A_96_p121697 | | 1.12 | 2.11 | MADS-box protein (AGL62) |
| A_96_p164501 | | 1.06 | 2.11 | CMT2 (CHROMOMETHYLASE 2) |
| A_96_p053756 | | 1.42 | 2.11 | SAP domain-containing protein |
| A_96_p068596 | | 1.22 | 2.11 | DNA-binding protein, putative |
| A_96_p025966 | | 1.60 | 2.11 | protein phosphatase 2C, putative |
| A_96_p004376 | | 1.39 | 2.11 | PEPKR2 |
| A_96_p148121 | | 1.36 | 2.11 | tyrosine specific protein phosphatase |
| A_96_p123097 | | 1.67 | 2.11 | kinesin motor protein-related |
| A_96_p104969 | | 1.54 | 2.11 | DIT1 (DICARBOXYLATE TRANSPORTER 1) |
| A_96_p148771 | | 0.99 | 2.11 | ABC transporter |
| A_96_p111462 | | 1.05 | 2.10 | ubiquitin-conjugating enzyme, e2 |
| A_96_p153561 | | 1.14 | 2.10 | zinc finger (C3HC4-type RING finger) |
| A_96_p104899 | | 1.20 | 2.10 | transferase |
| A_96_p106369 | | 1.78 | 2.10 | CER1 protein, putative (WAX2) |
| A_96_p011691 | | 1.88 | 2.10 | calreticulin 2 (CRT2) |
| A_96_p152706 | | 1.44 | 2.10 | glycosyltransferase 1 |
| A_96_p038016 | | 1.35 | 2.10 | glycine-rich protein |
| A_96_p121812 | | 1.38 | 2.10 | GRF5 (GROWTH-REGULATING FACTOR 5) |
| A_96_p066601 | | 1.22 | 2.10 | DEAD/DEAH box helicase, putative |
| A_96_p039406 | | 1.43 | 2.10 | dna-3-methyladenine glycosylase |
| A_96_p168914 | | 0.99 | 2.10 | ribosomal protein L7Ae |
| A_96_p009436 | | 1.65 | 2.10 | 60S ribosomal protein L26 (RPL26A) |
| A_96_p222234 | | 0.95 | 2.10 | subtilase |
| A_96_p159911 | | 0.66 | 2.10 | pollen-specific protein sf3, putative |
| A_96_p236673 | | 0.98 | 2.10 | dicarboxylate/tricarboxylate carrier (DTC) |
| A_96_p237387 | | 1.33 | 2.10 | ABC transporter |
| A_96_p170605 | | 1.58 | 2.08 | auxin-responsive protein, putative |
| A_96_p095329 | | 1.45 | 2.08 | SBP1 (S-RIBONUCLEASE BINDING PROTEIN 1) |
| A_96_p130467 | | 1.48 | 2.08 | CHLOROPLAST-LOCALIZED ISCA-LIKE PROTEIN) |
| A_96_p202174 | | 0.78 | 2.08 | calmodulin-binding |
| A_96_p228099 | | 1.63 | 2.08 | pectinesterase |
| A_96_p073634 | | 0.97 | 2.08 | similar to pathogenesis-related protein sth-2 |
| A_96_p112897 | | 1.06 | 2.08 | glycosyltransferase (UGT72B1) |
| A_96_p194794 | | 1.46 | 2.08 | glycosyl hydrolase 17 protein |
| A_96_p231054 | | 1.55 | 2.08 | Cytochrome 450 81D5 (CYP81D5) |
| A_96_p209319 | | 1.44 | 2.08 | NRPA2 (nuclear RNA polymerase A 2) |
| A_96_p127397 | | 1.76 | 2.08 | RWP-RK domain-containing protein |
| A_96_p108977 | | 0.93 | 2.08 | DNA binding |
| A_96_p172864 | | 1.32 | 2.08 | MTSSB (mitochondrially targeted single-stranded DNA binding protein) |
| A_96_p172474 | | 0.89 | 2.08 | phox (PX) domain-containing protein |
| A_96_p200229 | | 1.00 | 2.08 | protein kinase |
| A_96_p222619 | | 0.80 | 2.08 | protein kinase |
| A_96_p239018 | | 0.87 | 2.08 | zinc finger (C3HC4-type RING finger) |
| A_96_p012821 | | 1.15 | 2.08 | skip5-like protein |
| A_96_p258242 | | 1.27 | 2.08 | GTP-binding protein-related |
| A_96_p086044 | | 1.13 | 2.08 | RNA recognition motif (RRM)-containing protein |
| A_96_p118232 | | 1.87 | 2.08 | pinhead protein (PINHEAD) |
| A_96_p054316 | | 1.12 | 2.07 | pectinesterase |
| A_96_p012421 | | 1.43 | 2.07 | aspartate carabmoyltransferase, chloroplast |
| A_96_p247722 | | 0.78 | 2.07 | putative ac transposase |
| A_96_p201724 | | 1.55 | 2.07 | protein kinase, putative |
| A_96_p024811 | | 1.48 | 2.07 | MAPKKK21 (protein kinase) |
| A_96_p199464 | | 1.40 | 2.07 | dual specificity protein phosphatase (DsPTP1) |
| A_96_p072559 | | 1.77 | 2.07 | phosphoinositide 5-phosphatase |
| A_96_p166899 | | 1.32 | 2.07 | nodulin |
| A_96_p047816 | | 1.44 | 2.07 | sugar transporter, putative |
| A_96_p113152 | | 1.06 | 2.07 | Putative RING-H2 finger protein |
| A_96_p176369 | | 1.50 | 2.06 | heat shock protein-related |
| A_96_p118397 | | 1.16 | 2.06 | alliinase |
| A_96_p116667 | | 1.77 | 2.06 | nitrogen regulation |
| A_96_p018331 | | 1.92 | 2.06 | acidic endochitinase (CHIB1) |
| A_96_p189179 | | 1.58 | 2.06 | transcriptional activator, putative |
| A_96_p038441 | | 1.28 | 2.06 | RNA binding |
| A_96_p166299 | | 1.30 | 2.06 | nuclear transport factor 2 (NTF2) |
| A_96_p063436 | | 1.41 | 2.06 | TOM22-V (TRANSLOCASE OUTER MITOCHONDRIAL MEMBRANE 22-V) |
| A_96_p064366 | | 1.76 | 2.06 | poly (ADP-ribose) polymerase, putative |
| A_96_p237632 | | 1.88 | 2.06 | ubiquitin activating enzyme e1 |
| A_96_p241614 | | 1.63 | 2.06 | RabGAP/TBC domain-containing protein |
| A_96_p192589 | | 1.07 | 2.06 | cyclin-dependent protein kinase |
| A_96_p119127 | | 0.76 | 2.06 | syntaxin-41 |
| A_96_p126597 | | 1.58 | 2.06 | SPL12 (SQUAMOSA PROMOTER-BINDING PROTEIN-LIKE 12) |
| A_96_p210389 | | 1.14 | 2.06 | heavy-metal-associated domain-containing protein |
| A_96_p081294 | | 0.98 | 2.06 | peroxidase, putative |
| A_96_p153586 | | 1.05 | 2.04 | heat shock protein 90 |
| A_96_p090654 | | 0.67 | 2.04 | CRCK3 (calmodulin-binding receptor-like cytoplasmic kinase 3) |
| A_96_p236233 | | 0.99 | 2.04 | homeobox-leucine zipper protein |
| A_96_p084679 | | 0.77 | 2.04 | FDH (FIDDLEHEAD) |
| A_96_p260552 | | 1.67 | 2.04 | GDH2 (GLUTAMATE DEHYDROGENASE 2) |
| A_96_p016176 | | 0.75 | 2.04 | diaminopimelate epimerase |
| A_96_p256552 | | 1.91 | 2.04 | branched-chain-amino-acid transaminase |
| A_96_p140472 | | 1.63 | 2.04 | disease resistance protein |
| A_96_p213004 | | 1.05 | 2.04 | L-ascorbate peroxidase 2 |
| A_96_p078899 | | 1.12 | 2.04 | glutamine amidotransferase |
| A_96_p171714 | | 1.06 | 2.04 | adenosine-deaminase |
| A_96_p212879 | | 0.88 | 2.04 | glycosyl transferase 1 protein |
| A_96_p175129 | | 0.94 | 2.04 | protein arginine N-methyltransferase |
| A_96_p050642 | | 1.00 | 2.04 | Cytochrome P450 81F4 (CYP81F4) |
| A_96_p170499 | | 1.22 | 2.04 | transcription initiation factor |
| A_96_p027321 | | 1.32 | 2.04 | PAPA-1-like |
| A_96_p058461 | | 1.54 | 2.04 | replication control protein, putative |
| A_96_p103704 | | 1.12 | 2.04 | transcriptional factor B3 |
| A_96_p033131 | | 1.45 | 2.04 | aspartyl-tRNA synthetase, putative |
| A_96_p094884 | | 1.00 | 2.04 | importin beta-2, putative |
| A_96_p165091 | | 0.93 | 2.04 | subtilase |
| A_96_p022276 | | 1.05 | 2.04 | EMB2458 (EMBRYO DEFECTIVE 2458) |
| A_96_p124387 | | 1.57 | 2.04 | antiporter/ drug transporte |
| A_96_p123522 | | 1.44 | 2.04 | hAT dimerisation domain-containing protein |
| A_96_p067226 | | 1.66 | 2.04 | zinc finger (C3HC4-type RING finger) |
| A_96_p152261 | | 1.10 | 2.03 | FPS1 (FARNESYL DIPHOSPHATE SYNTHASE 1) |
| A_96_p231479 | | 1.48 | 2.03 | S-locus lectin protein kinase |
| A_96_p175424 | | 1.77 | 2.03 | far-red impaired responsive protein, putative |
| A_96_p055446 | | 1.63 | 2.03 | pathogenesis-related thaumatin |
| A_96_p027661 | | 1.89 | 2.03 | catalase 2 |
| A_96_p162066 | | 1.88 | 2.03 | cytidine/deoxycytidylate deaminase |
| A_96_p154201 | | 0.88 | 2.03 | glycosyl transferase 1 protein |
| A_96_p040266 | | 1.04 | 2.03 | NPH4 (NON-PHOTOTROPHIC HYPOCOTYL); transcription factor |
| A_96_p086484 | | 1.07 | 2.03 | Mzinc finger (GATA type) |
| A_96_p024841 | | 1.18 | 2.03 | heat shock factor protein, putative (HSF6) |
| A_96_p153661 | | 1.91 | 2.03 | SNF2 domain-containing protein |
| A_96_p125957 | | 1.12 | 2.03 | fertilization-independent endosperm protein |
| A_96_p155046 | | 1.18 | 2.03 | atp-dependent rna helicase-like protein |
| A_96_p018266 | | 0.77 | 2.03 | ribosomal protein S5 |
| A_96_p066311 | | 0.89 | 2.03 | 60S ribosomal protein L37a (RPL37aB) |
| A_96_p146181 | | 1.88 | 2.03 | CPK28 (calcium-dependent protein kinase 28) |
| A_96_p182644 | | 1.64 | 2.03 | fimbrin-like protein, putative |
| A_96_p108947 | | 1.66 | 2.03 | ABC transporter |
| A_96_p184819 | | 0.94 | 2.03 | polypeptide with a gag-like domain |
| A_96_p182709 | | 1.03 | 2.03 | RNA recognition motif (RRM)-containing protein |
| A_96_p124387 | | 1.31 | 2.04 | antiporter/ drug transporte |
| A_96_p123522 | | 1.66 | 2.04 | hAT dimerisation domain-containing protein |
| A_96_p067226 | | 1.55 | 2.04 | zinc finger (C3HC4-type RING finger) |
| A_96_p152261 | | 1.76 | 2.03 | FPS1 (FARNESYL DIPHOSPHATE SYNTHASE 1) |
| A_96_p231479 | | 1.16 | 2.03 | S-locus lectin protein kinase |
| A_96_p175424 | | 1.28 | 2.03 | far-red impaired responsive protein, putative |
| A_96_p055446 | | 1.14 | 2.03 | pathogenesis-related thaumatin |
| A_96_p027661 | | 0.88 | 2.03 | catalase 2 |
| A_96_p162066 | | 0.92 | 2.03 | cytidine/deoxycytidylate deaminase |
| A_96_p154201 | | 1.22 | 2.03 | glycosyl transferase 1 protein |
| A_96_p040266 | | 0.87 | 2.03 | NPH4 (NON-PHOTOTROPHIC HYPOCOTYL); transcription factor |
| A_96_p086484 | | 0.67 | 2.03 | Mzinc finger (GATA type) |
| A_96_p024841 | | 1.07 | 2.03 | heat shock factor protein, putative (HSF6) |
| A_96_p153661 | | 1.25 | 2.03 | SNF2 domain-containing protein |
| A_96_p125957 | | 1.87 | 2.03 | fertilization-independent endosperm protein |
| A_96_p155046 | | 1.05 | 2.03 | atp-dependent rna helicase-like protein |
| A_96_p018266 | | 0.93 | 2.03 | ribosomal protein S5 |
| A_96_p066311 | | 1.89 | 2.03 | 60S ribosomal protein L37a (RPL37aB) |
| A_96_p146181 | | 1.44 | 2.03 | CPK28 (calcium-dependent protein kinase 28) |
| A_96_p182644 | | 1.04 | 2.03 | fimbrin-like protein, putative |
| A_96_p108947 | | 1.85 | 2.03 | ABC transporter |
| A_96_p184819 | | 0.68 | 2.03 | polypeptide with a gag-like domain |
| A_96_p182709 | | 0.79 | 2.03 | RNA recognition motif (RRM)-containing protein |
| A_96_p124387 | | 1.12 | 2.04 | antiporter/ drug transporte |
| A_96_p102774 | | 1.69 | 2.01 | leucine-rich repeat transmembrane protein kinase, putative |
| A_96_p220024 | | 1.97 | 2.01 | QD17 (IQ-domain 17); calmodulin |
| A_96_p185919 | | 1.77 | 2.01 | histone H2B |
| A_96_p001481 | | 1.56 | 2.01 | disease resistance protein (NBS-LRR class), putative |
| A_96_p113762 | | 1.09 | 2.01 | protein disulfide isomerase |
| A_96_p019601 | | 0.87 | 2.01 | phosphoribosylformylglycinamidine synthase |
| A_96_p100059 | | 0.99 | 2.01 | galactosyl transferase GMA12/MNN10 |
| A_96_p198779 | | 1.90 | 2.01 | myb transcription factor |
| A_96_p231934 | | 1.78 | 2.01 | mitochondrial transcription termination factor-related / mTERF-related |
| A_96_p140652 | | 1.34 | 2.01 | nucleic acid binding protein |
| A_96_p205449 | | 1.85 | 2.01 | pseudouridine synthase |
| A_96_p061596 | | 0.87 | 2.01 | protein kinase |
| A_96_p256302 | | 0.86 | 2.01 | PP2A-4 (protein phosphatase 2A-4) |
| A_96_p077449 | | 0.79 | 2.01 | peptidase M3 |
| A_96_p191589 | | 1.09 | 2.01 | kinesin motor protein-related |
| A_96_p165846 | | 1.11 | 2.01 | ROF1 (ROTAMASE FKBP 1) |
| A_96_p246462 | | 1.54 | 2.01 | UPL1 (UBIQUITIN-PROTEIN LIGASE 1) |
| A_96_p129012 | | 1.32 | 2.00 | leucine-rich repeat transmembrane protein kinase, putative |
| A_96_p214239 | | 1.08 | 2.00 | HAT22 (homeobox-leucine zipper protein 22); |
| A_96_p064191 | | 1.06 | 2.00 | FUT12 (fucosyltransferase 12) |
| A_96_p085609 | | 0.98 | 2.00 | nitrate reductase [nadh] |
| A_96_p246427 | | 1.06 | 2.00 | GRF5 (GROWTH-REGULATING FACTOR 5) |
| A_96_p121497 | | 1.22 | 2.00 | pentatricopeptide (PPR) repeat-containing protein |
| A_96_p124327 | | 1.10 | 2.00 | ASHH3 (HISTONE-LYSINE N-METHYLTRANSFERASE ASHH3 |
| A_96_p049621 | | 0.98 | 2.00 | ATP binding / protein kinase |
| A_96_p080129 | | 1.13 | 2.00 | ubiquitin thiolesterase/ zinc ion binding |
| A_96_p160076 | | 1.65 | 2.00 | zinc finger (C3HC4-type RING finger) |
| A_96_p063176 | | 1.43 | 2.00 | WD-40 repeat protein |
| A_96_p234329 | | 1.23 | 2.00 | no apical meristem (NAM) (NAC2) |
| A_96_p043161 | | 1.87 | 2.00 | MATE efflux protein-related |
| A_96_p245900 | | 1.05 | 2.00 | AXR1 (AUXIN RESISTANT 1) |
| **Roots of tomato plants treated with either EM 0.1 or EM 1 mL L^-1^: *up-regulation by both treatments*** | | | | |
| **Agilent ID** | | **Fold change** | | **Annotation** |
|  | | *EM 0.1 mL L^-1^* | *EM 1 mL L^-1^* |  |
| A_96_p181989 | | 7.14 | 3.72 | cell wall-associated hydrolase |
| A_96_p045476 | | 6.91 | 17.89 | Glutathione S-transferase |
| A_96_p011406 | | 6.65 | 2.69 | aldo/keto reductase |
| A_96_p181024 | | 6.09 | 2.43 | Heat shock protein 91 |
| A_96_p187884 | | 5.94 | 2.31 | Chitinase |
| A_96_p151561 | | 5.28 | 6.98 | calcium-dependent protein kinase 33 |
| A_96_p206279 | | 5.25 | 2.36 | aldo/keto reductase |
| A_96_p059781 | | 4.86 | 3.29 | ribosomal protein L7A |
| A_96_p102244 | | 4.52 | 2.32 | multidrug resistance-associated protein 6 |
| A_96_p246567 | | 4.09 | 18.34 | Chitinase |
| A_96_p108952 | | 4.05 | 5.23 | calcium-dependent protein kinase 33 |
| A_96_p155271 | | 3.94 | 21.21 | alcohol dehydrogenase 1 |
| A_96_p087979 | | 3.94 | 2.06 | Malate dehydrogenase (NAD), mitochondrial |
| A_96_p113532 | | 3.51 | 2.24 | OPR2, 12-oxophytodienoate reductase 2 |
| A_96_p225804 | | 3.34 | 2.21 | OLEO1 (Oleosin1) |
| A_96_p150911 | | 3.33 | 3.41 | hemoglobin class 1 |
| A_96_p068421 | | 3.31 | 3.33 | Protein Pir |
| A_96_p004906 | | 3.29 | 2.02 | Peroxidase N1 |
| A_96_p227069 | | 3.26 | 2.97 | receptor kinases leucine rich repeat II |
| A_96_p247132 | | 3.21 | 2.62 | BHLH038/ORG2 (OBP3-RESPONSIVE GENE 2) |
| A_96_p054591 | | 3.12 | 11.63 | chromosome chr8 scaffold_23, transcription factor |
| A_96_p184439 | | 3.09 | 2.41 | basic helix-loop-helix (bHLH) |
| A_96_p188834 | | 3.00 | 2.04 | DNAJ heat shock N-terminal domain-containing protein |
| A_96_p118662 | | 2.95 | 2.46 | TIR1, Transport Inhibitor Response 1 |
| A_96_p220014 | | 2.92 | 2.29 | Vacuolar protein sorting-associated protein 35 |
| A_96_p136212 | | 2.90 | 3.66 | Heat shock protein 81-1 |
| A_96_p016146 | | 2.87 | 2.09 | putative yellow stripe-like protein |
| A_96_p077514 | | 2.85 | 2.2 | Cytochrome P450, CYP94B1 |
| A_96_p041071 | | 2.85 | 2.16 | translation initiation factor 6 (eif-6)-like protein |
| A_96_p131782 | | 2.85 | 13.36 | chromosome chr8 scaffold_23, transcription factor |
| A_96_p074814 | | 2.84 | 3.82 | purple acid phosphatase 27 |
| A_96_p206574 | | 2.84 | 2.22 | NGA1 transcription factor |
| A_96_p183174 | | 2.75 | 2.05 | anaphase-promoting complex/cyclosome 8 |
| A_96_p043431 | | 2.73 | 85.78 | Expansin-Like B1 |
| A_96_p138737 | | 2.65 | 2.23 | (SERK2) somatic embryogenesis receptor-like kinase 2 |
| A_96_p223239 | | 2.61 | 2.35 | DNAJ heat shock N-terminal domain-containing protein (J20) |
| A_96_p019616 | | 2.59 | 2.08 | SRG1 (senescence-related gene 1) |
| A_96_p232879 | | 2.58 | 6.42 | kelch repeat-containing protein type 1 |
| A_96_p066161 | | 2.57 | 2.19 | Nucleoporin NUP1 |
| A_96_p018746 | | 2.51 | 2.37 | Kelch repeat-containing protein |
| A_96_p237427 | | 2.47 | 2.77 | glutamate receptor (GLR3.5) |
| A_96_p161991 | | 2.46 | 2.06 | DREB2A (DRE-BINDING PROTEIN 2A) |
| A_96_p008426 | | 2.44 | 9.55 | Universal stress protein (USP) |
| A_96_p071044 | | 2.41 | 2.04 | sec61beta |
| A_96_p079489 | | 2.32 | 3.46 | SRG1, Senescence-related gene 1 |
| A_96_p163051 | | 2.32 | 2.32 | cathepsin B-like cysteine protease, putative |
| A_96_p038446 | | 2.31 | 2.41 | heat shock transcription factor B2A |
| A_96_p099674 | | 2.29 | 2.11 | Heat shock protein 101 |
| A_96_p246285 | | 2.17 | 4.26 | Histone H2A-like protein |
| A_96_p221699 | | 2.16 | 3.01 | ATCAT6/CAT6 (CATIONIC AMINO ACID TRANSPORTER 6) |
| A_96_p177699 | | 2.12 | 2.15 | Notchless-like protein |
| A_96_p040196 | | 2.12 | 7.73 | Gibberellin 3 beta-hydroxylase |
| A_96_p089644 | | 2.12 | 2.77 | 1-aminocyclopropane-1-carboxylate oxidase 4 (ACC oxidase) |
| A_96_p155256 | | 2.10 | 3.04 | EFE, Ethylene forming enzyme |
| A_96_p167029 | | 2.10 | 2.02 | sucrose-phosphate synthase isoform c |
| A_96_p171729 | | 2.09 | 2.10 | Cytochrome P450, CYP94C1 |
| A_96_p247297 | | 2.08 | 2.65 | purple acid phosphatase 15 |
| A_96_p236018 | | 2.07 | 3.22 | UDP-glucoronosyl/UDP-glucosyl transferase |
| A_96_p115977 | | 2.06 | 2.28 | late blight resistance protein, putative |
| A_96_p097479 | | 2.06 | 2.64 | isoflavone reductase, putative |
| A_96_p081064 | | 2.05 | 2.69 | UDP-glycosyltransferase 73B3 |
| A_96_p232634 | | 2.04 | 2.42 | glutamate receptor (GLR3.5) |
| A_96_p017141 | | 2.03 | 4.31 | LBD41 (LOB DOMAIN-CONTAINING PROTEIN 41) |
| A_96_p201344 | | 2.03 | 2.28 | ATPDR9/PDR9 (PLEIOTROPIC DRUG RESISTANCE 9) |
| A_96_p225839 | | 2.01 | 4.78 | AMP-dependent synthetase and ligase |
| A_96_p145996 | | 2.01 | 3.67 | CER1 (ECERIFERUM 1) |
| A_96_p230747 | | 1.97 | 2.82 | ERF1-3, translation release factor |
| A_96_p051021 | | 1.92 | 2.04 | BTB-POZ and MATH domain 4 |
| **Roots of tomato plants treated with either EM 0.1 or EM 1 mL L^-1^: *down-regulation by both treatments*** | | | | |
| **Agilent ID** | | **Fold change** | | **Annotation** |
|  | | *EM 0.1 mL L^-1^* | *EM 1 mL L^-1^* |  |
| A_96_p028556 | | 0.01 | 0.36 | phosphatidylinositol 3- and 4-kinase |
| A_96_p028556 | | 0.01 | 0.36 | phosphatidylinositol 3- and 4-kinase |
| A_96_p146426 | | 0.02 | 0.36 | Ethylene-responsive transcription factor CRF4 |
| A_96_p228839 | | 0.10 | 0.36 | protease-associated zinc finger (C3HC4-type RING finger) |
| A_96_p120607 | | 0.11 | 0.36 | transducin / WD-40 repeat |
| A_96_p226109 | | 0.11 | 0.20 | very-long-chain fatty acid condensing enzyme, putative |
| A_96_p055872 | | 0.20 | 0.41 | GDSL-motif lipase/hydrolase |
| A_96_p186619 | | 0.18 | 0.38 | thiol-disulfide exchange intermediate |
| A_96_p221829 | | 0.20 | 0.41 | chloride channel-like (CLC) protein, putative |
| A_96_p185834 | | 0.21 | 0.39 | BRC2/TCP12 (BRANCHED2); transcription factor |
| A_96_p006366 | | 0.23 | 0.38 | senescence/dehydration-associated protein-related |
| A_96_p054441 | | 0.26 | 0.39 | AP2 domain-containing protein |
| A_96_p116462 | | 0.22 | 0.32 | histidine kinase (AHK4) (WOL) |
| A_96_p032626 | | 0.29 | 0.39 | DND1 (DEFENSE NO DEATH 1) |
| A_96_p208944 | | 0.33 | 0.41 | glutamyl-tRNA(Gln) amidotransferase, putative |
| A_96_p227009 | | 0.30 | 0.30 | CKX3 (CYTOKININ OXIDASE 3) |
| A_96_p034856 | | 0.31 | 0.37 | Cytochrome P450 71A22 (CYP71A20) |
| A_96_p107614 | | 0.31 | 0.38 | UDP-glucoronosyl/UDP-glucosyl transferase |
| A_96_p017641 | | 0.31 | 0.26 | ICE1 (INDUCER OF CBF EXPRESSION 1) |
| A_96_p107934 | | 0.31 | 0.38 | kinase |
| A_96_p190659 | | 0.31 | 0.39 | auxin response factor 10 |
| A_96_p160891 | | 0.32 | 0.32 | polyphenol oxidase e, chloroplast precursor |
| A_96_p204919 | | 0.32 | 0.32 | CMT3 (CHROMOMETHYLASE 3) |
| A_96_p152376 | | 0.34 | 0.40 | Cytochrome P450 71A22 (CYP71A22) |
| A_96_p045641 | | 0.35 | 0.39 | mitochondrial substrate carrier |
| A_96_p006671 | | 0.35 | 0.38 | hydroxymethylbilane synthase |
| A_96_p176424 | | 0.35 | 0.40 | CER1 protein |
| A_96_p123187 | | 0.36 | 0.39 | ribonuclease III |
| A_96_p066211 | | 0.37 | 0.39 | gibberellin-regulated |
| A_96_p188479 | | 0.37 | 0.38 | myrcene/(E)-beta-ocimene synthase |
| A_96_p053536 | | 0.40 | 0.43 | pfkB-type carbohydrate kinase |
| A_96_p046976 | | 0.43 | 0.43 | copper chaperone (CCH)-related |
| A_96_p100054 | | 0.43 | 0.43 | CAC2 (acetyl co-enzyme A carboxylase biotin carboxylase subunit) |
| **Roots of tomato plants treated with either EM 0.1 or EM 1 mL L^-1^: *up-regulation in EM0.1 and down-regulation or no effect in EM1*** | | | | |
| **Agilent ID** | | **Fold change** | | **Annotation** |
|  | | *EM 0.1 mL L^-1^* | *EM 1 mL L^-1^* |  |
| A_96_p186839 | | 10.18 | 1.46 | leucine-rich repeat |
| A_96_p005086 | | 8.21 | 0.96 | oxidoreductase, zinc-binding dehydrogenase |
| A_96_p107514 | | 7.84 | 1.84 | PHD zinc finger |
| A_96_p157751 | | 6.08 | 1.32 | responsive to abscisic acid 1B (RAB1B) |
| A_96_p203739 | | 5.11 | 1.35 | glycosyl hydrolase 3 protein |
| A_96_p188139 | | 4.89 | 0.38 | gibberellin-responsive protein 5 (GASA5) |
| A_96_p219814 | | 4.58 | 1.83 | ribosomal protein L11 |
| A_96_p052751 | | 4.39 | 1.94 | elongation factor Tu |
| A_96_p113102 | | 4.27 | 1.64 | transferase |
| A_96_p155621 | | 4.25 | 1.76 | acyl-(acyl-carrier-protein) desaturase, putative |
| A_96_p134332 | | 4.13 | 1.66 | phototropic-responsive NPH3 |
| A_96_p143326 | | 4.11 | 0.4 | gibberellin-responsive protein 5 (GASA5) |
| A_96_p217964 | | 4.05 | 0.92 | pectinesterase |
| A_96_p158521 | | 3.91 | 0.95 | Cytochrome P450 78A6 (CYP78A6) |
| A_96_p119187 | | 3.81 | 0.97 | Pathogenesis-related protein Bet v |
| A_96_p191069 | | 3.78 | 1.92 | Ca^2+^-dependent lipid-binding protein (CLB1) |
| A_96_p067906 | | 3.73 | 1.82 | nodule-enhanced protein phosphatase type 2C |
| A_96_p147216 | | 3.71 | 0.95 | ST (steroid sulfotransferase) |
| A_96_p160246 | | 3.65 | 0.97 | peroxidase, putative |
| A_96_p193869 | | 3.63 | 1.93 | anther-specific protein lat52 precursor |
| A_96_p175819 | | 3.61 | 1.32 | putative senescence-associated protein |
| A_96_p212484 | | 3.51 | 0.87 | EMB2753 (EMBRYO DEFECTIVE 2753) |
| A_96_p112297 | | 3.31 | 0.97 | histone H3.2 protein |
| A_96_p185354 | | 3.17 | 0.96 | auxin-responsive factor (ARF9) |
| A_96_p172219 | | 3.08 | 1.52 | acetyl-CoA C-acyltransferase |
| A_96_p161186 | | 2.97 | 1.54 | CUT1 (CUTICULAR 1) |
| A_96_p053992 | | 2.88 | 1.54 | putative auxin-induced saur-like protein |
| A_96_p005561 | | 2.8 | 1.6 | RAN GTPase activating protein 2 (RanGAP2) |
| A_96_p114702 | | 2.76 | 1.82 | protein kinase |
| A_96_p025176 | | 2.73 | 1.9 | DEAH box helicase, putative |
| A_96_p220949 | | 2.73 | 1.96 | disease resistance protein RPS2 |
| A_96_p109207 | | 2.73 | 1.95 | sulfate transporter 2.1 |
| A_96_p187919 | | 2.68 | 0.9 | Bet v I allergen , similar to Csf-2 |
| A_96_p231314 | | 2.65 | 1.75 | caffeoyl-CoA 3-O-methyltransferase, putative |
| A_96_p009126 | | 2.64 | 1.76 | ribosomal protein L36 |
| A_96_p087949 | | 2.58 | 1.36 | putative permease I |
| A_96_p153221 | | 2.55 | 0.96 | CDP-related, similar to CCAAT displacement protein (CDP) (Cut-like 1) |
| A_96_p001491 | | 2.55 | 1.56 | calmodulin-binding |
| A_96_p192154 | | 2.54 | 1.45 | histone H2B, putative |
| A_96_p103199 | | 2.54 | 1.41 | zinc finger (GATA type) |
| A_96_p209074 | | 2.53 | 1.87 | heat shock protein 98.7 |
| A_96_p069509 | | 2.53 | 1.67 | DNAJ heat shock N-terminal domain-containing protein |
| A_96_p181189 | | 2.52 | 0.41 | terpene synthase/cyclase |
| A_96_p058016 | | 2.52 | 1.86 | zinc transporter (ZIP2) |
| A_96_p203494 | | 2.51 | 0.97 | EIF3A (eukaryotic translation initiation factor 3A) |
| A_96_p016476 | | 2.5 | 1.74 | OPP7 (Type one serine/threonine protein phosphatase 7) |
| A_96_p132047 | | 2.49 | 0.93 | auxin-responsive |
| A_96_p182089 | | 2.48 | 1.3 | copper transporter, putative |
| A_96_p047671 | | 2.45 | 1.65 | acyl CoA reductase, putative |
| A_96_p226599 | | 2.45 | 0.86 | putative polyprotein |
| A_96_p122422 | | 2.45 | 1.44 | putative inward rectifying potassium channel |
| A_96_p112552 | | 2.44 | 0.98 | protein kinase |
| A_96_p213399 | | 2.41 | 1.51 | DNA repair protein RAD54 |
| A_96_p181814 | | 2.41 | 1.5 | DNAJ heat shock protein, putative |
| A_96_p246522 | | 2.4 | 0.99 | 17.6 kDa class I heat shock protein (HSP17.6A-CI) |
| A_96_p151431 | | 2.37 | 0.87 | SNF2 domain-containing protein |
| A_96_p110017 | | 2.37 | 1.85 | indole-3-acetic acid induced-related |
| A_96_p144946 | | 2.36 | 1.44 | zinc finger (C2H2 type) |
| A_96_p073919 | | 2.35 | 0.97 | pectinesterase ppe8b precursor |
| A_96_p141122 | | 2.35 | 1.55 | guanine nucleotide-binding |
| A_96_p121252 | | 2.34 | 1.45 | carotene 7,8-desaturase |
| A_96_p089399 | | 2.34 | 1.22 | Heat shock protein 81-2 (HSP81-2) |
| A_96_p170529 | | 2.33 | 0.97 | protein kinase |
| A_96_p213364 | | 2.32 | 1.75 | importin beta-2 subunit |
| A_96_p101519 | | 2.32 | 1.56 | flavodoxin |
| A_96_p175519 | | 2.31 | 1.45 | elongation factor 1-alpha |
| A_96_p016101 | | 2.3 | 1.46 | putative serine decarboxylase |
| A_96_p062436 | | 2.3 | 1.25 | Cytochrome P450 76G1 (CYP76G1) |
| A_96_p141042 | | 2.28 | 1.75 | MRH5/SHV3 (morphogenesis of root hair 5) |
| A_96_p160316 | | 2.28 | 1.54 | thioredoxin |
| A_96_p206324 | | 2.27 | 0.39 | iron-phytosiderophore transporter protein yellow stripe 1 |
| A_96_p134792 | | 2.27 | 1.52 | S-adenosyl-L-homocysteine hydrolase, putative |
| A_96_p158126 | | 2.27 | 1.58 | calcium-binding EF hand |
| A_96_p127582 | | 2.25 | 0.99 | serine hydroxymethyltransferase 4 |
| A_96_p161731 | | 2.24 | 0.95 | Cytochrome P450 86A1(CYP86A1) |
| A_96_p077029 | | 2.24 | 1.76 | Cytochrome P450 94B1 (CYP94B1) |
| A_96_p053521 | | 2.24 | 1.23 | protein transporter |
| A_96_p151316 | | 2.24 | 1.88 | DDT domain-containing protein |
| A_96_p245410 | | 2.24 | 1.2 | Heat shock protein 101 |
| A_96_p221969 | | 2.23 | 1.43 | elongation factor Tu |
| A_96_p001431 | | 2.22 | 0.95 | glycyl-tRNA synthetase |
| A_96_p226209 | | 2.21 | 1.06 | ARP (apurinic endonuclease-redox protein) |
| A_96_p176154 | | 2.21 | 1.86 | ADP, ATP carrier protein 1, mitochondrial |
| A_96_p197729 | | 2.21 | 1.66 | monoterpene synthase 1 |
| A_96_p097054 | | 2.2 | 1.32 | putative zinc transporter |
| A_96_p047306 | | 2.18 | 1.1 | pectinesterase |
| A_96_p176429 | | 2.16 | 0.98 | elongation factor 1-alpha |
| A_96_p166274 | | 2.16 | 0.98 | homeobox-leucine zipper protein |
| A_96_p179804 | | 2.14 | 0.38 | nitrate transporter (NTP2) |
| A_96_p053506 | | 2.13 | 1.34 | AP2 domain-containing transcription factor, putative |
| A_96_p237387 | | 2.13 | 1.32 | ABC transporter |
| A_96_p160381 | | 2.11 | 1.86 | peroxidase, putative |
| A_96_p106349 | | 2.1 | 1.22 | ATTIC110 (translocon at the inner envelope membrane of chloroplasts 110) |
| A_96_p171154 | | 2.09 | 0.95 | MLO4 (MILDEW RESISTANCE LOCUS O 4) |
| A_96_p067741 | | 2.08 | 0.96 | phosphatidic acid phosphatase alpha |
| A_96_p007931 | | 2.08 | 1.64 | eukaryotic translation initiation factor 3 subunit 3 |
| A_96_p022701 | | 2.08 | 1.78 | basic helix-loop-helix (bHLH) |
| A_96_p165196 | | 2.07 | 1.85 | polyphenol oxidase d |
| A_96_p198084 | | 2.07 | 1.55 | auxin-induced saur-like protein |
| A_96_p113807 | | 2.07 | 1.5 | 60S ribosomal protein L23A |
| A_96_p094779 | | 2.06 | 1.8 | phenylalanyl-tRNA synthetase class IIc |
| A_96_p132357 | | 2.05 | 1.44 | isoleucyl-tRNA synthetase, putative |
| A_96_p216194 | | 2.04 | 1.38 | dehydroquinate dehydratase |
| A_96_p030701 | | 2.04 | 1.44 | ABC transporter |
| A_96_p018991 | | 2.03 | 1.36 | GRF3 (GROWTH-REGULATING FACTOR 3) |
| A_96_p193114 | | 2.03 | 1.46 | zinc finger (C2H2 type) |
| A_96_p236248 | | 2.03 | 1.44 | Ethylene-insensitive protein 2 (EIN2) |
| A_96_p248192 | | 2.03 | 1.42 | proton-dependent oligopeptide transport (POT) |
| A_96_p124662 | | 2.02 | 0.95 | protein arginine N-methyltransferase, putative |
| A_96_p134962 | | 2.02 | 1.68 | LOB domain protein 4 |
| A_96_p219604 | | 2.01 | 1.48 | expansin, putative (EXP8) |
| A_96_p025756 | | 2.01 | 1.64 | TPS10 (terpene synthase 10) |
| A_96_p157646 | | 2.01 | 0.89 | protein kinase |
| A_96_p116062 | | 2.01 | 1.36 | purine transmembrane transporter |
| A_96_p226759 | | 2.01 | 0.96 | wall-associated kinase, putative |
| A_96_p030741 | | 2.01 | 1.12 | sulfate transmembrane transporter 3.1 |
| A_96_p107449 | | 2.00 | 1.18 | ATP:ADP antiporter |
| A_96_p112317 | | 2.00 | 1.91 | leucine-rich repeat transmembrane protein kinase, putative |
| Roots of tomato plants treated with either EM 0.1 mL L^-1^ or EM 1 mL L^-1^: ***Up-regulation in EM1 and down-regulation or no effect in EM0.1*** | | | | |
| **Agilent ID** | | **Fold change** | | **Annotation** |
|  | | *EM 0.1 mL L^-1^* | *EM 1 mL L^-1^* |  |
| A_96_p146486 | | 0.93 | 80.48 | ZIFL1 (ZINC INDUCED FACILITATOR-LIKE 1) |
| A_96_p180714 | | 1.94 | 52.72 | Organic cation/carnitine transporter 2 |
| A_96_p138582 | | 1.27 | 33.34 | Cytochrome P450 94B2 (CYP94B2) |
| A_96_p017671 | | 1.82 | 33.07 | IAA-amido synthases |
| A_96_p007546 | | 1.6 | 28.46 | Cytochrome P450 86A7 (CYP86A7) |
| A_96_p050501 | | 1.85 | 27.51 | LOB domain protein 41 |
| A_96_p228739 | | 1.36 | 25.69 | pentatricopeptide (PPR) repeat-containing protein |
| A_96_p200144 | | 0.9 | 15.6 | diacylglycerol kinase, putative |
| A_96_p146881 | | 1.54 | 12.96 | calmodulin-domain protein kinase isoform 9 (CPK9) |
| A_96_p004906 | | 0.35 | 11.27 | family II extracellular lipase 1 (EXL1) |
| A_96_p156096 | | 1.77 | 11.17 | alcohol dehydrogenase 2 |
| A_96_p154161 | | 1.68 | 10.69 | alcohol dehydrogenase 2 |
| A_96_p206539 | | 1.34 | 10.3 | hydroxy-3-methylglutaryl coenzyme A reductase |
| A_96_p113632 | | 1.14 | 7.04 | UDP-glucoronosyl/UDP-glucosyl transferase |
| A_96_p049386 | | 1.08 | 6.82 | pyruvate decarboxylase, putative |
| A_96_p025311 | | 1.08 | 6.72 | curculin-like (mannose-binding) lectin |
| A_96_p020911 | | 1.65 | 6.44 | DNAJ heat shock N-terminal domain-containing protein |
| A_96_p072339 | | 0.33 | 6.43 | CNGC1 (cyclic nucleotide-gated channel 1) |
| A_96_p017276 | | 1.56 | 6.43 | 2OG-Fe(II) oxygenase |
| A_96_p196584 | | 1.83 | 6.27 | ATPase, coupled to transmembrane movement of substances |
| A_96_p247757 | | 1.9 | 6.02 | amino acid transporter |
| A_96_p118762 | | 1.85 | 5.67 | peroxidase, putative |
| A_96_p054796 | | 1.76 | 5.44 | ER small heat shock protein (HSP22.0-ER) |
| A_96_p077019 | | 1.82 | 5.42 | Cytochrome P450 72A15 (CYP72A15) |
| A_96_p233959 | | 1.98 | 5.38 | serine/threonine-protein kinase |
| A_96_p044086 | | 1.56 | 5.29 | zinc finger (C2H2 type) |
| A_96_p197884 | | 0.96 | 5.18 | amino acid transporter |
| A_96_p044476 | | 1.33 | 5.17 | WRKY DNA-binding protein 18 |
| A_96_p107864 | | 1.54 | 5.11 | aspartate aminotransferase APS1 |
| A_96_p021771 | | 0.95 | 5.07 | zinc finger (C3HC4-type RING finger) |
| A_96_p163836 | | 0.89 | 5.03 | MYB78 (myb domain protein 78) |
| A_96_p225819 | | 1.54 | 4.99 | aldo/keto reductase, putative |
| A_96_p012601 | | 0.87 | 4.86 | ACS2 (1-Amino-cyclopropane-1-carboxylate synthase 2) |
| A_96_p197494 | | 0.98 | 4.84 | zinc-binding dehydrogenase |
| A_96_p115027 | | 1.34 | 4.75 | amino acid transporter |
| A_96_p031306 | | 1.94 | 4.68 | UDP-glucoronosyl/UDP-glucosyl transferase |
| A_96_p231399 | | 0.87 | 4.68 | ARA12 subtilase |
| A_96_p040721 | | 1.32 | 4.58 | F-box |
| A_96_p007831 | | 1.68 | 4.57 | acyl-(acyl-carrier-protein) desaturase, putative |
| A_96_p172359 | | 1.91 | 4.54 | CBL-interacting protein kinase 25 |
| A_96_p119457 | | 1.72 | 4.31 | AMP-dependent synthetase and ligase |
| A_96_p009201 | | 1.87 | 4.29 | polygalacturonase, putative |
| A_96_p045291 | | 1.14 | 4.23 | MYB96 (myb domain protein 96) |
| A_96_p113192 | | 1.87 | 4.2 | Mitogen-activated protein kinase kinase kinase 3 |
| A_96_p058846 | | 1.65 | 4.2 | transferase |
| A_96_p057921 | | 0.37 | 4.16 | terpene synthase |
| A_96_p126097 | | 1.18 | 4.14 | Expansin-like B1 |
| A_96_p074924 | | 1.75 | 4.13 | glutamine-dependent asparagine synthetase 1 (ASN1) |
| A_96_p022771 | | 1.37 | 4.11 | leucine-rich repeat transmembrane protein kinase, putative |
| A_96_p143916 | | 1.22 | 4.11 | zinc finger (C3HC4-type RING finger) |
| A_96_p107769 | | 1.94 | 4.04 | auxin-responsive |
| A_96_p214054 | | 1.76 | 4.04 | anthocyaninless2 (ANL2) |
| A_96_p177974 | | 1.76 | 4.02 | expressed protein, similar to auxin down-regulated protein ARG10 |
| A_96_p143966 | | 1.26 | 3.97 | heavy-metal-associated domain-containing protein |
| A_96_p086494 | | 1.87 | 3.96 | RAV transcription factor |
| A_96_p132732 | | 1.04 | 3.95 | auxin/aluminum-responsive protein, putative |
| A_96_p121427 | | 0.89 | 3.89 | subtilase |
| A_96_p124987 | | 1.12 | 3.8 | AAA-type ATPase |
| A_96_p260947 | | 0.99 | 3.8 | CER1 protein |
| A_96_p026456 | | 0.04 | 3.72 | HSP70B (heat shock protein 70B) |
| A_96_p108522 | | 1.78 | 3.71 | IAA-amido synthases |
| A_96_p043716 | | 1.26 | 3.7 | coatomer protein complex, subunit beta 2 (beta prime), putative |
| A_96_p173669 | | 0.84 | 3.61 | 4-hydroxyphenylpyruvate dioxygenase (4HPPD) |
| A_96_p044356 | | 1.11 | 3.59 | thioredoxin |
| A_96_p201016 | | 1.43 | 3.58 | LOX5 lipoxygenase |
| A_96_p094614 | | 1.04 | 3.56 | aspartate aminotransferase APS2 |
| A_96_p146941 | | 1.05 | 3.56 | purple acid phosphatase 27 |
| A_96_p245145 | | 1.04 | 3.49 | 2OG-Fe(II) oxygenase |
| A_96_p248092 | | 1.87 | 3.47 | Allene oxide synthase |
| A_96_p167694 | | 1.6 | 3.47 | soluble starch synthase 1 |
| A_96_p193269 | | 1.43 | 3.31 | carbonate dehydratase 1 (CA1) |
| A_96_p201224 | | 1.9 | 3.27 | PHD finger transcription factor, putative |
| A_96_p008131 | | 0.95 | 3.25 | Rho-GTPase-activating protein-related |
| A_96_p064191 | | 1.24 | 3.22 | HAT22 (homeobox-leucine zipper protein 22) |
| A_96_p151571 | | 1.12 | 3.19 | Peroxidase 2 (PER2) |
| A_96_p044396 | | 1.34 | 3.19 | AAA-type ATPase |
| A_96_p012961 | | 1.12 | 3.18 | ELI3-1 (ELICITOR-ACTIVATED GENE 3) |
| A_96_p128672 | | 1.48 | 3.16 | wound-induced protein win2 precursor |
| A_96_p109882 | | 1.87 | 3.15 | ATPP2-B15 (Phloem protein 2-B15) |
| A_96_p229144 | | 1.24 | 3.09 | glycosyl transferase 2 protein |
| A_96_p021311 | | 1.65 | 3.09 | eucine-rich repeat transmembrane protein kinase, putative |
| A_96_p090874 | | 1.08 | 3.07 | Peroxidase 12 (PER12) |
| A_96_p062051 | | 1.18 | 3.05 | armadillo/beta-catenin repeat |
| A_96_p030681 | | 0.88 | 3.02 | ethylene-responsive transcription factor 2 |
| A_96_p200099 | | 1.08 | 3.01 | glucan endo-1,3-beta-glucosidase b precursor |
| A_96_p030001 | | 1.06 | 3 | glutamate-tRNA ligase, putative |
| A_96_p246512 | | 1.57 | 2.98 | heat shock transcription factor A6B |
| A_96_p160346 | | 1.96 | 2.91 | synthase |
| A_96_p169004 | | 1.03 | 2.91 | zinc finger (C5HC2 type) |
| A_96_p077869 | | 0.87 | 2.88 | calcineurin-like phosphoesterase |
| A_96_p245090 | | 1.84 | 2.87 | CAT2 (catalase 2) |
| A_96_p098569 | | 1.45 | 2.87 | prephenate dehydrogenase |
| A_96_p113612 | | 1.23 | 2.86 | ferric-chelate reductase/oxidoreductase |
| A_96_p153111 | | 1.15 | 2.82 | disease resistance protein (TIR-NBS-LRR class), putative |
| A_96_p033111 | | 1.36 | 2.8 | monooxygenase |
| A_96_p212529 | | 1.55 | 2.79 | MATE efflux protein-related |
| A_96_p070104 | | 1.65 | 2.78 | proton-dependent oligopeptide transport (POT) |
| A_96_p141177 | | 1.13 | 2.77 | U-box domain-containing protein |
| A_96_p032731 | | 1.22 | 2.73 | ankyrin repeat |
| A_96_p019601 | | 1.14 | 2.73 | protein disulfide isomerase-like (PDIL) protein |
| A_96_p227264 | | 1.78 | 2.72 | WAK2 (wall-associated kinase 2) |
| A_96_p062496 | | 1.21 | 2.71 | calmodulin-binding protein |
| A_96_p138797 | | 1.2 | 2.66 | tropinone reductase, putative |
| A_96_p065726 | | 1.3 | 2.65 | calmodulin-binding protein |
| A_96_p201369 | | 1.23 | 2.64 | SOUL heme-binding |
| A_96_p051031 | | 1.62 | 2.52 | proton-dependent oligopeptide transport (POT) |
| A_96_p108517 | | 0.96 | 2.5 | alpha-N-acetylglucosaminidase |
| A_96_p173249 | | 1.32 | 2.48 | enolase |
| A_96_p040341 | | 1.09 | 2.42 | ethylene-forming enzyme (ACO) |
| A_96_p091460 | | 1.8 | 2.4 | glutathione S-transferase |
| A_96_p061786 | | 1.47 | 2.4 | MORN (Membrane Occupation and Recognition Nexus) repeat-containing protein |
| A_96_p163356 | | 1.06 | 2.39 | mitogen-activated protein kinase 4 |
| A_96_p016501 | | 0.87 | 2.37 | PTR3 (PEPTIDE TRANSPORTER PROTEIN 3) |
| A_96_p226054 | | 1.55 | 2.37 | ABC transporter |
| A_96_p130807 | | 0.16 | 2.35 | dehydrodolichyl diphosphate synthase, putative |
| A_96_p073634 | | 1.18 | 2.31 | pathogenesis-related protein sth-2 |
| A_96_p202479 | | 0.21 | 2.29 | auxin efflux carrier |
| A_96_p177374 | | 1.44 | 2.27 | cycloartenol synthase |
| A_96_p093284 | | 1.54 | 2.26 | phosphoenolpyruvate carboxylase |
| A_96_p255102 | | 0.93 | 2.26 | Ran-binding protein 1 domain-containing protein |
| A_96_p134872 | | 0.25 | 2.25 | glycerophosphoryl diester phosphodiesterase |
| A_96_p078364 | | 1.24 | 2.25 | glutathione S-transferase |
| A_96_p165791 | | 1.54 | 2.25 | phosphate-responsive protein, putative |
| A_96_p078364 | | 1.89 | 2.25 | glutathione S-transferase |
| A_96_p158141 | | 1.35 | 2.24 | mitogen-activated protein kinase 3 |
| A_96_p226479 | | 0.95 | 2.22 | ethylene receptor, putative (EIN4) |
| A_96_p072125 | | 1.83 | 2.22 | glutathione S-transferase |
| A_96_p001466 | | 0.36 | 2.21 | leucine-rich repeat |
| A_96_p227384 | | 1.08 | 2.21 | glutamate receptor (GLR2.1) |
| A_96_p229549 | | 0.22 | 2.2 | CNGC1 (cyclic nucleotide-gated channel 1) |
| A_96_p091654 | | 1.28 | 2.2 | ATPAO4 (polyamine oxidase 4) |
| A_96_p125497 | | 1.86 | 2.19 | disease resistance protein (NBS-LRR class), putative |
| A_96_p221584 | | 1.82 | 2.19 | myosin heavy chain MYA2 |
| A_96_p145521 | | 1.71 | 2.18 | phosphoenolpyruvate carboxylase kinase 2 (PPCK2) |
| A_96_p175979 | | 1.66 | 2.17 | disease resistance protein (CC-NBS-LRR class), putative |
| A_96_p104361 | | 1.66 | 2.17 | Glutamine synthetase cytosolic isozyme |
| A_96_p144591 | | 1.45 | 2.15 | myo-inositol oxygenase |
| A_96_p055586 | | 0.99 | 2.15 | potassium channel tetramerisation domain-containing protein |
| A_96_p033886 | | 1.41 | 2.14 | glutathione S-transferase 6 |
| A_96_p189739 | | 0.34 | 2.12 | S-adenosyl-l-methionine:salicylic acid carboxyl methyltransferase |
| A_96_p123682 | | 1.22 | 2.1 | cellulose synthase-like E1 |
| A_96_p070214 | | 1,58 | 2.1 | ABA-responsive protein (HVA22a) |
| A_96_p044341 | | 1.8 | 2.08 | disease resistance protein (CC-NBS-LRR class), putative |
| A_96_p112857 | | 1.66 | 2.07 | leucine-rich repeat |
| A_96_p211104 | | 1.34 | 2.06 | early-responsive to dehydration stress ERD3 protein |
| A_96_p144606 | | 1.32 | 2.06 | PAL1 phenylalanine ammonia-lyase |
| A_96_p097229 | | 1.26 | 2.05 | delta-OAT (ornithine- delta-aminotransferase) |
| A_96_p015986 | | 0.98 | 2.05 | osmotic stress-responsive proline dehydrogenase, putative |
| A_96_p209869 | | 1.83 | 2.01 | phosphate transporter (PT2) |
| A_96_p123757 | | 1.87 | 2.01 | HKT1 (high affinity K+ transporter 1) |
| A_96_p191269 | | 1.53 | 2.01 | cysteine proteinase, putative |
